# Pulmonary Atresia With Intact Septum Survival, Reintervention, and Days in Hospital Through Childhood: Contemporary Outcomes From a National Registry

**DOI:** 10.1161/CIRCINTERVENTIONS.125.016435

**Published:** 2026-08-14

**Authors:** Dan-Mihai Dorobantu, Qi Huang, Ferran Espuny-Pujol, Katherine L. Brown, Sok-Leng Kang, Asmita Singhania, Rodney C. Franklin, Sonya Crowe, Christina Pagel, Serban C. Stoica

**Affiliations:** 1 Children’s Health and Exercise Research Centre (CHERC), Medical School, University of Exeter, United Kingdom (D.-M.D.).; 2 Departments of Cardiology and Cardiac Surgery, Bristol NHS Foundation Trust, United Kingdom (D.-M.D., A.S., S.C.S.).; 3 Clinical Operational Research Unit, Department of Mathematics, University College London 4919 , United Kingdom (Q.H., F.E.-P., S.C., C.P.).; 4 Department of Computer Science, University of Reading, United Kingdom (F.E.-P.).; 5 Heart and Lung Division, Great Ormond Street Hospital NIHR Biomedical Research Centre, London, United Kingdom (K.L.B.).; 6 Department of Cardiology, Alder Hey Children’s NHS Foundation Trust, Liverpool, United Kingdom (S.-L.K.).; 7 Department of Paediatric Cardiology, Royal Brompton and Harefield National Health Service Foundation Trust, London, United Kingdom (R.C.F.).; 8 Bristol Medical School, University of Bristol, United Kingdom (S.C.S.).

**Keywords:** cardiac surgical procedures, heart defects, congenital, length of stay, pulmonary atresia, risk factors

## Abstract

**BACKGROUND:**

Pulmonary atresia with intact ventricular septum management has evolved, with increased use of transcatheter right ventricular decompression, but the impact on contemporary outcomes remains unknown. This study evaluates contemporary national outcomes in patients aged 0 to 18 years.

**METHODS:**

Patients with pulmonary atresia with intact ventricular septum undergoing cardiac intervention (2000–2022) in England and Wales were linked to multiple national data sets. Mortality (early and late), reintervention, and hospital stay were described overall and by treatment pathway.

**RESULTS:**

Among 488 included patients with pulmonary atresia with intact ventricular septum, 58.8% underwent right ventricular decompression as the first procedure, predominantly transcatheter (84.8%), while 32.4% received stage 1 palliation (arteriopulmonary shunt or ductal stenting), and 8.8% died or were lost to follow-up before either intervention. Overall, 54.5% achieved biventricular (BiV) repair, 26.7% underwent functionally single-ventricle palliation, and 18.8% were noncommitted. Overall group in-hospital mortality was 11.7%. The median follow-up was 8.9 years, with 1- and 5-year mortality of 19.8% (95% CI, 16.2%–23.2%) and 20.8% (95% CI, 17.1%–24.4%), respectively. Stage 1 palliation as the initial therapy had higher 1-year mortality than right ventricular decompression (37.2% [29.0%–44.5%] versus 7.7% [4.5%–10.7%]). Cardiac reintervention was 62.6% (56.1%–68.4%) at 10 years after BiV repair and 32.8% (21.5%–44.4%) at 10 years after palliative stage 3. Median hospital stay was greatest in the first year (35 days), declining to 2 days annually by 10 years, with functionally single-ventricle patients requiring more days in hospital than BiV patients (52 versus 28).

**CONCLUSIONS:**

In a contemporary pulmonary atresia with intact ventricular septum cohort, transcatheter right ventricular decompression was the predominant initial intervention. This approach had lower in-hospital mortality compared with older multicenter series, with BiV repair achieved in more than half of the patients. Early mortality after nonantegrade flow augmentation remains high, likely due to nonmodifiable risk factors in severe cases. Long-term survival and hospital stay remain better for those amenable to BiV circulation, reflecting more favorable anatomy compared with those on the single-ventricle pathway, although reintervention is lower in the latter.

**What is Known**

- Pulmonary atresia with intact ventricular septum management involves complex treatment decisions resulting in biventricular or single-ventricle pathways.
- Transcatheter interventions to augment pulmonary blood flow are emerging as important alternatives to traditional arterial shunt surgery.
- Overall outcomes remain poor, especially in the first year of life and for patients with the most severe forms of the disease.

**What the Study Adds**

- In a modern UK cohort, more than half of all patients achieved biventricular repair with transcatheter right ventricular decompression being the predominant first procedure.
- Early mortality after initial treatment has decreased compared with older, nonselected patient series, reflecting a positive impact of changes in management.

Pulmonary atresia with intact ventricular septum (PA-IVS) encompasses a spectrum of right ventricular (RV) and pulmonary valve hypoplasia.^1^ Treatment pathways include biventricular repair (BiV), functionally single-ventricle (fSV) palliation, and less commonly, one-and-a-half-ventricle circulation or heart transplantation. Initial strategies to augment pulmonary perfusion range from surgical or transcatheter RV decompression to retrograde flow augmentation with arteriopulmonary shunts (APS) or ductal stenting.^1–5^

Nonmodifiable factors such as right ventricle-dependent coronary circulation (RVDCC) or severe right-sided hypoplasia may preclude right ventricular decompression (RVD) and BiV repair. Beyond these cases, initial management of PA-IVS varies across centers and has evolved over the past 2 decades from favoring APS toward more transcatheter RVD, though practice remains heterogeneous.^2,3,5–8^

The only 2 large PA-IVS registry reports, both from North America, illustrate the evolving management of PA-IVS.^9,10^ In the early 2000s, initial procedures were predominantly surgical RVD with APS or APS alone,^9^ later shifting toward RVD or patent ductus arteriosus (PDA) stenting, with APS still remaining common.^10^ Survival improved between the 2 eras. Contemporary single-center series support early RVD and transcatheter-first strategies as emerging standards,^1,3,6^ but a modern multicenter cohort confirming the impact of these changes on outcomes is not available.

The current study used data from the UK national audit registry and National Health Service records to report contemporary outcomes of PA-IVS surgical and transcatheter management from birth to adolescence by describing: (1) the initial intervention choices and treatment pathways; (2) early and late hospital mortality by first intervention type (stage 1 palliation or RVD) and treatment pathway (BiV and fSV); and (3) late reintervention types and hospital resource utilization (days spent in hospital) by treatment pathway (fSV or BiV). These data serve as a modern benchmark for PA-IVS treatment outcomes, while also shedding more light on any improvements in outcomes with evolving treatment strategies.

## Methods

### National Data Sets and Linkage

The National Congenital Heart Disease Audit from April 2000 to March 2022 is the core data set, with Office for National Statistics mortality data retrieved in August 2023. Submission of National Congenital Heart Disease Audit data is mandatory for each cardiac procedure case and is routinely quality controlled. Because of the sensitive nature of the data collected for this study, which includes national data sets, requests for data use can only be made to the National Health Service or Office for National Statistics.

A subset of National Congenital Heart Disease Audit data from April 2000 to March 2017 was also linked to several national healthcare data sets (Figure S1): Pediatric Intensive Care Audit Network for patient admissions to pediatric intensive care units (ICU); and Hospital Episode Statistics routine administrative data on inpatient, outpatient, and accident and emergency (A&E) care at hospitals in England. The linkage methodology and an overview of the data set have been described elsewhere.^11^ The study was approved by the North of Scotland National Health Service Research Ethics Committee on February 14, 2020 (number 20/NS/0022) and Health Research Authority Confidentiality Advisory Group on July 12, 2020 (number 20/Confidentiality Advisory Group/0027), permitting the use of registry data for specific research purposes without consent.

All clinical data were organized into care spells, which can contain procedures, inpatient or intensive care stays, outpatient or A&E visits, or any combination, with no >1 day between events.^11^ For each linked data set, the available data cover different time periods and outcomes, as described in Figure S1. The data extracted from the LAUNCHES QI (Linking Audit and National Data Sets in Congenital Heart Services for Quality Improvement) and CHAMPION (Congenital Heart Audit: Measuring Progress in Outcomes Nationally) data sets are detailed in the Supplemental Methods.

### Patient Selection and Classification

All patients with a diagnosis of PA-IVS, without more complex associated defects, undergoing any cardiac procedure (surgical or interventional) between April 2000 and March 2021 were included. These are detailed in Figure 1.

**Figure 1.**
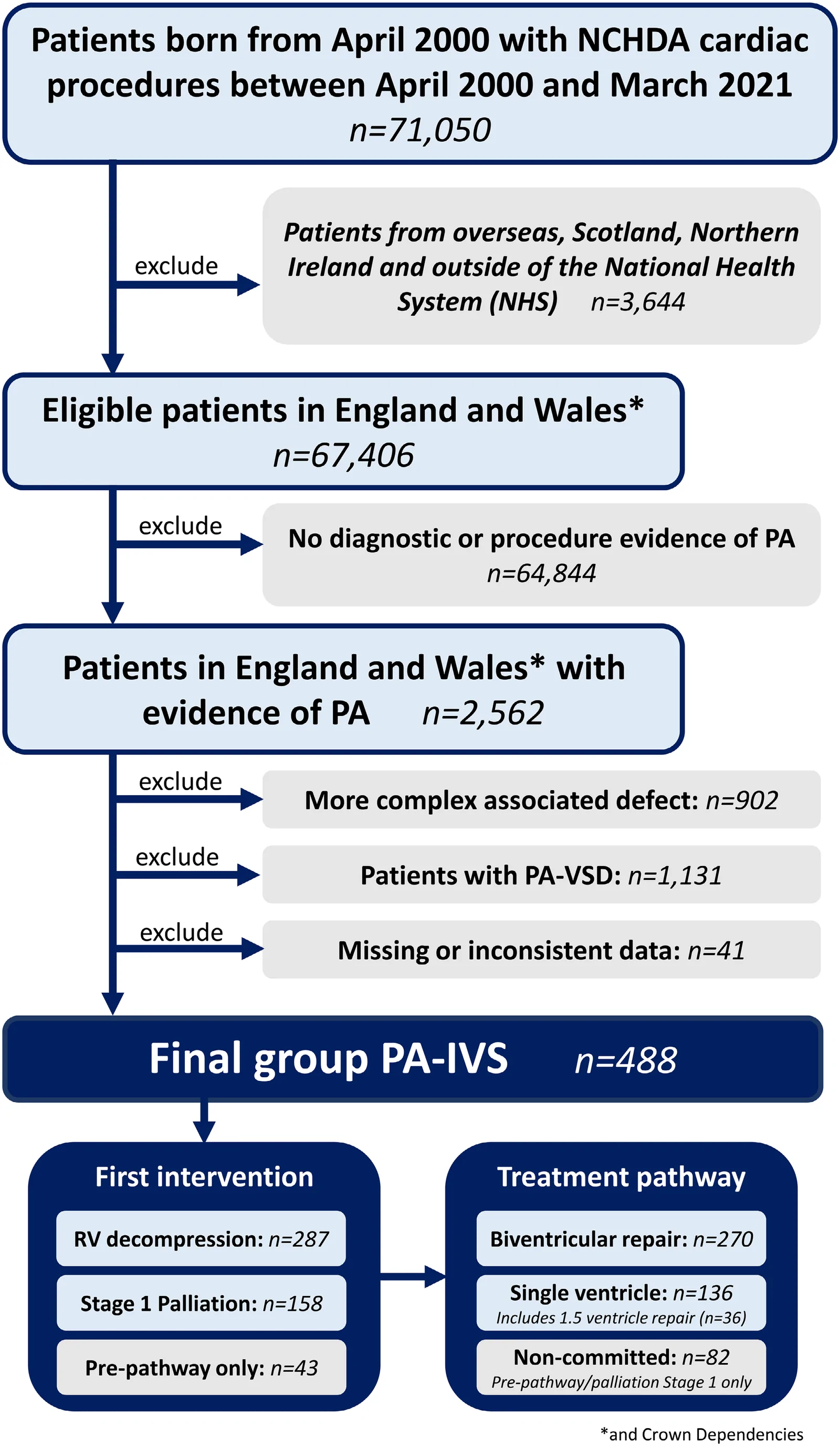
**Flowchart of included and excluded patient groups.** IVS indicates intact ventricular septum; NCHDA, National Congenital Heart Disease Audit; PA, pulmonary atresia; RV, right ventricular; and VSD, ventricular septal defect.

The corresponding diagnosis/procedure codes, specific procedure classifications,^12^ inclusion and exclusion criteria, and diagnosis/procedure type classifications are further detailed in the Supplemental Methods and Tables S1 through S4.

### Treatment Pathway

The following types of pathway procedures were defined (detailed in Table S5): (1) prepathway; (2) palliative stage 1, including APS and ductal stenting; (3) RVD; (4) palliative stage 2, including any procedure with superior cavopulmonary (Glenn) shunt; and (5) palliative stage 3, Fontan-type operation.

Depending on the treatment sequences, each patient was classified into the following mutually exclusive pathways (detailed in Table S5): (1) fSV, including 1.5-ventricle circulation; (2) BiV repair; and (3) noncommitted patients (neither BiV repair nor at least palliative stage 2).

### Collected Variables and Outcomes

The following data were collected from the linked national data sets:

- Patient-level data: sex, ethnicity, area of residence deprivation index, preterm birth, antenatal diagnosis, additional cardiac risk factors (associated cardiac conditions or complications not consisting of other defects, such as pulmonary hypertension, cardiac dysfunction, cardiomyopathy, and congenital comorbidity (noncardiac).
- Procedure-level data: age at procedure, weight at procedure, acquired comorbidity (the presence of an acquired complication related to congenital heart disease, eg, necrotizing enterocolitis, renal failure), severity of illness markers (such as circulatory support, shock, or acidosis),^16^ cardiac procedure type, procedure era, and center. These were previously defined as part of the Partial Risk Adjustment in Surgery model.^12^
- Hospital resource utilization: hospital length of stay and ICU length of stay for the first procedure spell, and from birth to the end of follow-up: any and cardiac-related inpatient and ICU stays and outpatient or A&E department visits.
- Mortality and reintervention: in relation to the first procedure or birth (clearly stated); early mortality and early cardiac reintervention (30-day and in-hospital), mid-term (1 and 5 years), and late (maximum follow-up) mortality and reintervention (any cardiac, surgical, transcatheter, and by anatomic subtype).

### Statistical Analysis

Frequencies are reported as counts and percentages (%), and continuous variables as medians (interquartile range [IQR]). Descriptive analyses included patient and procedure characteristics, treatment pathways, hospital resource utilization (inpatient, outpatient, and A&E visits), and mortality by Kaplan-Meier analysis. Median hospital resource utilization was summarized per month in the first year and annually thereafter, using all patients with available data for at least part of each period.

In-hospital mortality and mortality at 30 days were reported by first procedure and treatment pathway. The probability of reintervention (first occurrence) after the index RV decompression, palliative stage 2, and palliative stage 3 was calculated using cumulative incidence functions, considering death without reintervention as a competing event.^13^ We applied data disclosure control rules that suppress all counts from 1 to 5 (marked as *), allowing 0 to be shown.

Data management and statistical analyses were conducted using the Stata/MP 17.0 software (StataCorp LLC, College Station, TX) or R, version 4.4.1 (R Core Team, 2021, Vienna, Austria). The Sankey diagrams were created using SankeyMATIC (https://sankeymatic.com/).

## Results

A total of 488 PA-IVS patients who underwent their first cardiac procedure between April 2000 and March 2022 were included (Figure 1). The Table summarizes demographic, clinical, and procedural characteristics for the overall cohort and by treatment pathway (fSV or BiV). There were 93 interruptions of pregnancy, 13 miscarriages, and 27 patients in whom no intervention was performed; these were not considered for inclusion but are mentioned here for completeness.

**Table.**
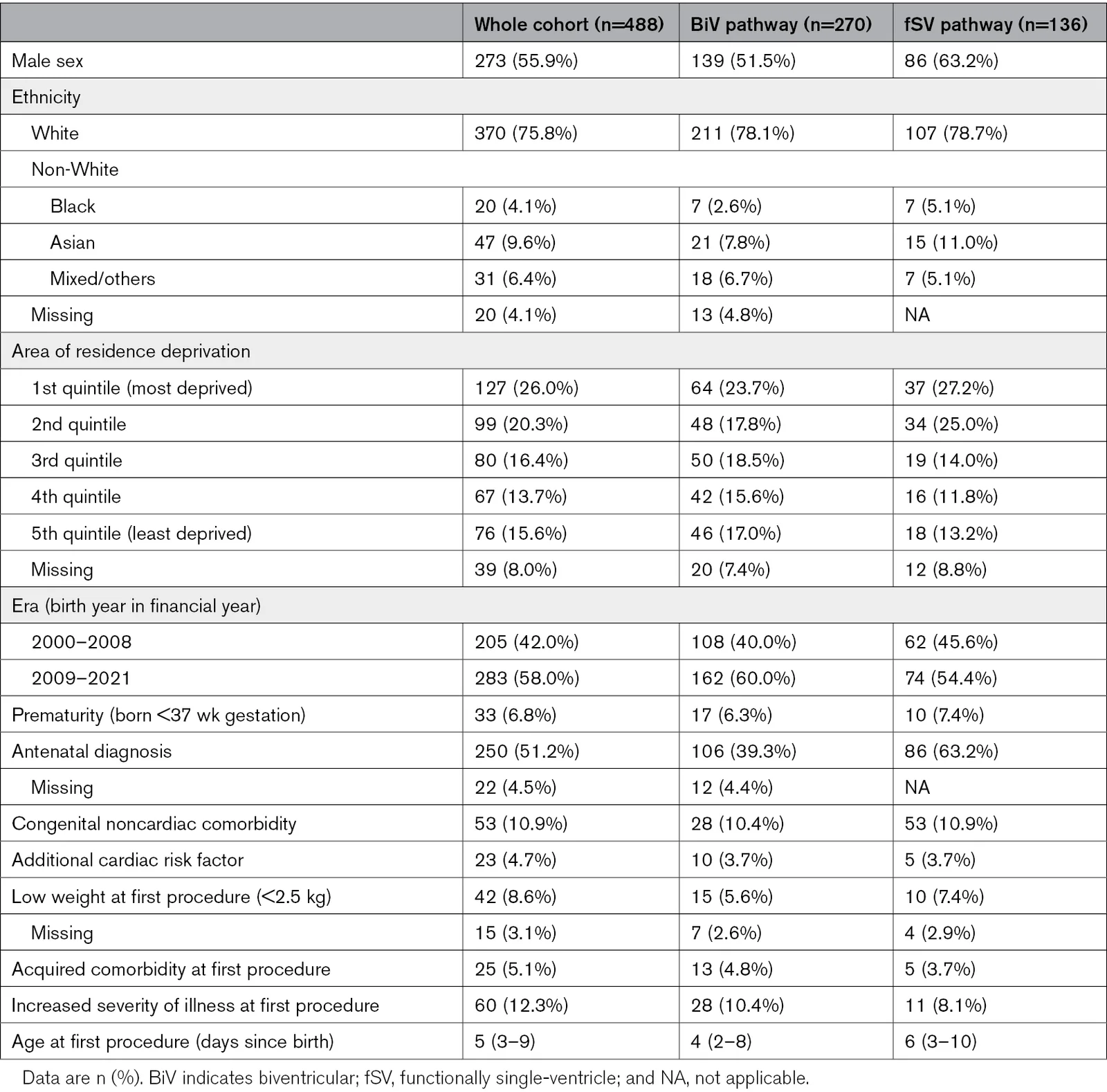
Patient Characteristics by Treatment Pathway

### PA-IVS Treatment Pathways

Figure 2 shows procedure pathway choices, mortality, and follow-up status. Of 488 patients, 287 (58.8%) underwent RVD as the first intervention, 152 (31.1%) received stage 1 palliation, and 43 (8.8%) had prepathway procedures such as atrial septostomy or pulmonary artery interventions.

**Figure 2.**
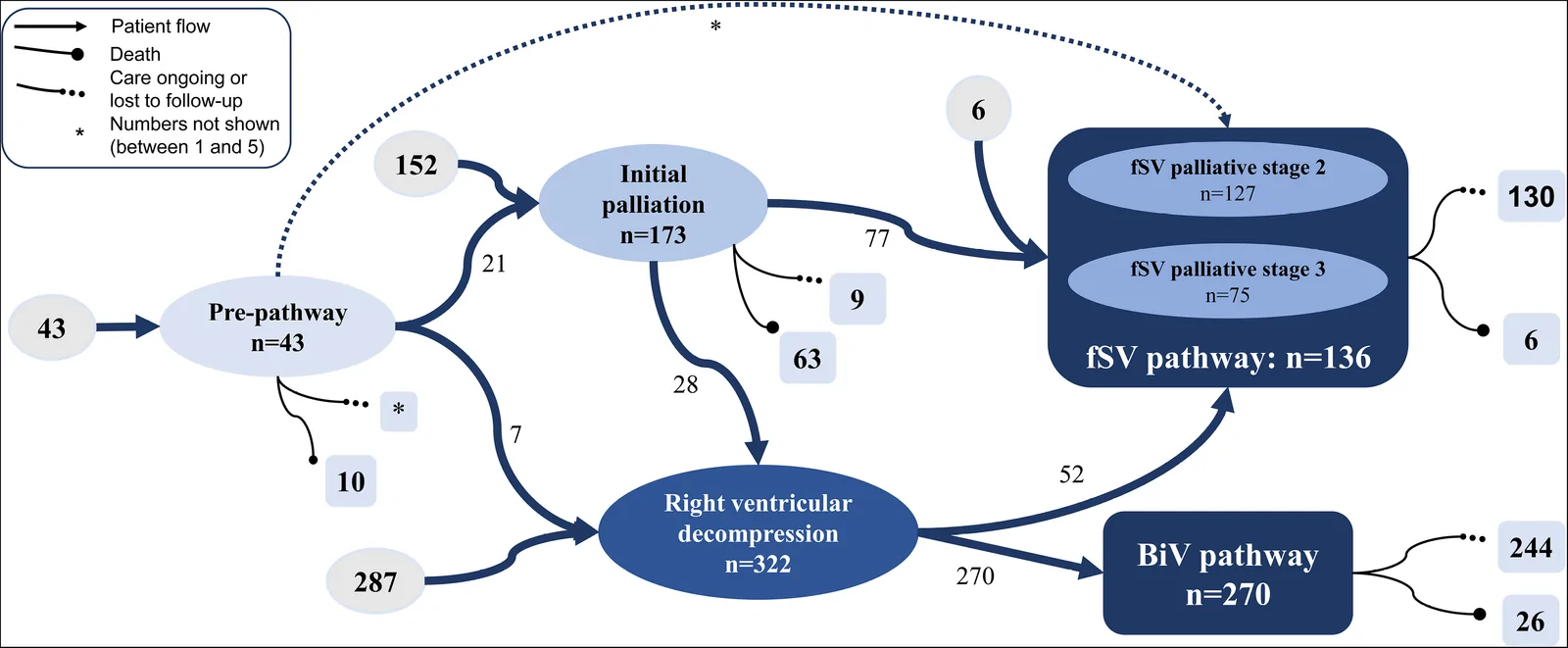
**Planned treatment pathways and mortality events for patients with pulmonary atresia and intact ventricular septum.** Continuous black line with a black circle at the end—death in hospital following a procedure; continuous black line with a white circle at the end—death after discharge; continuous purple line—transition to a care pathway procedure; interrupted purple line—ongoing care or lost to follow-up; asterisk—numbers not shown (between 1 and 5). BiV indicates biventricular; and fSV, functionally single-ventricle.

By treatment choice, 136 (27.9%) followed an fSV pathway, including 23 (4.7%) with one-and-a-half-ventricle circulation; 270 (55.3%) achieved BiV repair; and 82 (16.8%) remained noncommitted. The latter were all initially treated with stage 1 palliation and died or were lost to follow-up before either RVD or stage 2 palliation.

Across all pathway choices, of all 322 RVD procedures, 49 (15.2%) were surgical (median age, 13 days; IQR, 5–106) and 273 (82.7%) were transcatheter (median age, 4 days; IQR, 3–8). Among all 173 stage 1 palliations, 140 (80.9%) were APS and 33 (19.1%) were PDA stents.

There was a degree of variability between centers in terms of RVD versus stage 1 palliation, catheter versus surgical RVD, and APS versus PDA stent treatment choices, as evidenced in Table S6.

### Early Outcomes and ICU Resource Utilization by First Procedure

Neonates undergoing stage 1 palliation as the first procedure had higher 30-day and in-hospital mortality rates compared with those undergoing RVD, while hospital length of stay and ICU length of stay were similar (Table S7). Length of stay after transcatheter and surgical RVD was also similar, with a median of 13 days (IQR, 8–22) versus 12 days (IQR, 6–19), respectively.

### Late Mortality by First Procedure and Treatment Pathway

Over a median follow-up of 8.9 years (IQR, 2.0–16.3; maximum, 23.3 years), 105 deaths occurred, most (73/105) after prepathway or stage 1 procedures within the noncommitted group. The 1-year, 5-year, and 10-year mortality rates for the whole cohort were 19.8% (95% CI, 16.2%–23.2%), 20.8% (95% CI, 17.1%–24.4%), and 21.7% (95% CI, 17.9%–25.3%), respectively (Figure 3A).

**Figure 3.**
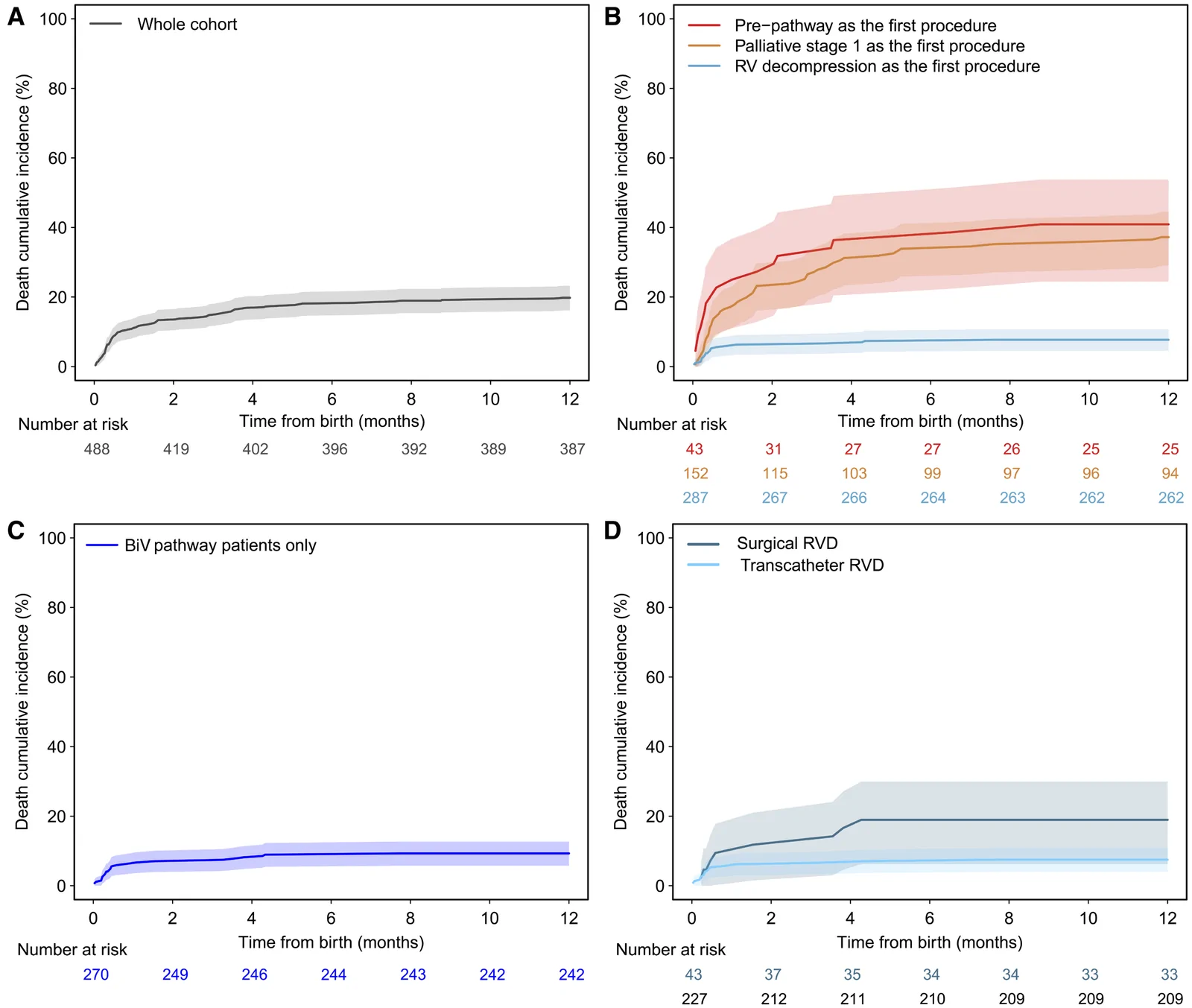
**Death rate curves (using Kaplan-Meier analysis) from birth to 1 year of age. A**, For the whole cohort overall. **B**, For the whole cohort by first procedure. **C**, For biventricular pathway (BiV) patients only. **D**, For BiV patients by right ventricular decompression (RVD) type. Patients who had stage 2 or 3 single-ventricle palliation (n=6) as the first procedure were not included in panel **B**. RV indicates right ventricular.

We further present the mortality curves before the age of 1 year for patients who underwent stage 1 palliation and those who underwent RV decompression without stage 1 palliation (Figure 3B). Patients undergoing stage 1 palliation had higher mortality than those with primary RV decompression, that is, 37.2% (95% CI, 29.0%–44.5%) versus 7.7% (95% CI, 4.6%–10.7%).

Among neonates on the BiV pathway (n=270), the median follow-up after index RVD was 10.4 years (IQR, 4.7–17.4; maximum, 23.3 years). There were 26 deaths, most within 30 days (19 of 26) and nearly all within the first year (96.2%, 25 of 26). The 1-year mortality was 9.3% overall (95% CI, 5.8%–12.7%), higher after surgical repair (19.8% [16.2%–23.2%]) than after catheter repair (7.5% [4.0%–10.9%]), as shown in Figure 3C and 3D.

### Cardiac Reinterventions by Treatment Pathway

Among 270 BiV pathway patients, 167 (61.9%) required at least 1 reintervention, first occurring at a median of 37 days after the RVD (IQR, 8–264), and 94 (34.8%) underwent multiple (≥2) reinterventions. Over the available median follow-up of 9.0 years (IQR, 3.4–16.0; maximum 22.0 years), reintervention rates were similar after surgical and catheter repair (62.8% versus 61.7%).

The cumulative incidence of cardiac reintervention rose steeply in the first year, reaching 51.1% (95% CI, 44.9%–57.0%; Figure 4A), then increased more gradually through 21 years (Figure 4C). Early reinterventions were driven mainly by PA/right ventricular outflow tract procedures and pulmonary blood flow augmentation (PDA stent, shunt, or septostomy; Figure 4B and 4D). Detailed probabilities are provided in Table S8. For patients on the fSV pathway, cardiac reinterventions were less common than in the BiV pathway. The cumulative incidence of cardiac reintervention at 5 years after stage 2 palliation (and before stage 3) was 24.4% (95% CI, 16.9%–32.6%), and at 10 years after stage 3 palliation was 32.8% (95% CI, 21.5%–44.4%), respectively (Figure 4E and 4F).

**Figure 4.**
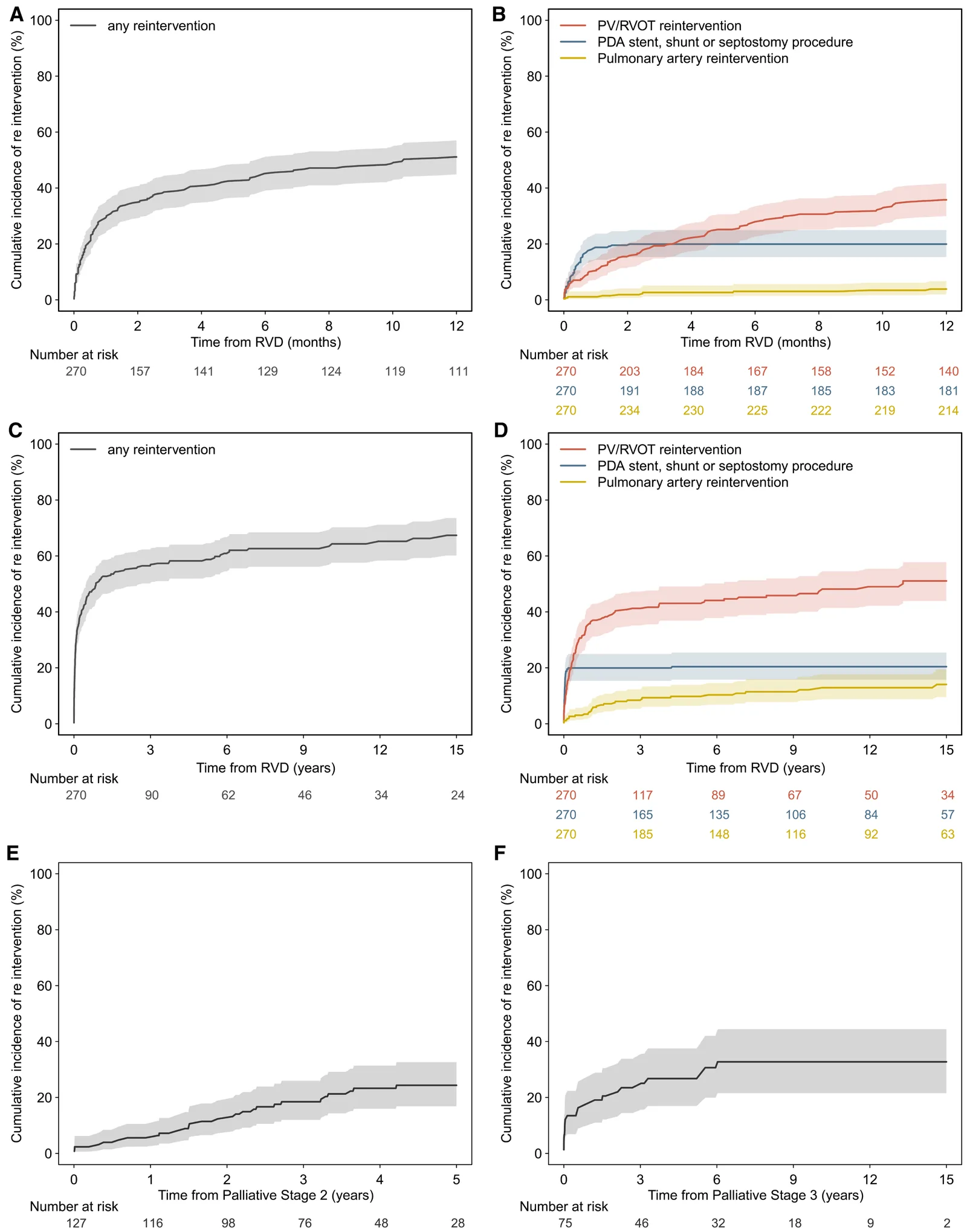
**Cardiac reintervention after the initial right ventricular decompression (RVD) procedure in biventricular repair (BiV) pathway and after stages 2 and 3 in the functionally single-ventricle (fSV) pathway. A**, BiV pathway—any cardiac reintervention in the first year of life. **B**, BiV pathway—cardiac reintervention in the first year of life by type. **C**, BiV pathway—any cardiac reintervention at maximum follow-up. **D**, BiV pathway—cardiac reintervention at maximum follow-up by type. **E**, fSV pathway—any cardiac reintervention after stage 2 palliation. **F**, fSV pathway—any cardiac reintervention after stage 3. Numerical values are detailed in Table S8. PDA indicates patent ductus arteriosus; PV, pulmonary valve; and RVOT, right ventricular outflow tract.

The subtypes of reinterventions and their sequence for BiV patients are presented in Figure 5A. Interstage and post-stage 2 reinterventions in fSV pathway patients are shown in Figure 5B through 5D. The detailed subtypes of reinterventions and the time of occurrence from the index RV decompression are presented in Table S9. These are detailed in a previous publication.^14^ As expected, forward-flow augmentation was uncommon in the fSV pathway, while procedures to further enhance nonantegrade pulmonary blood flow were common.

**Figure 5.**
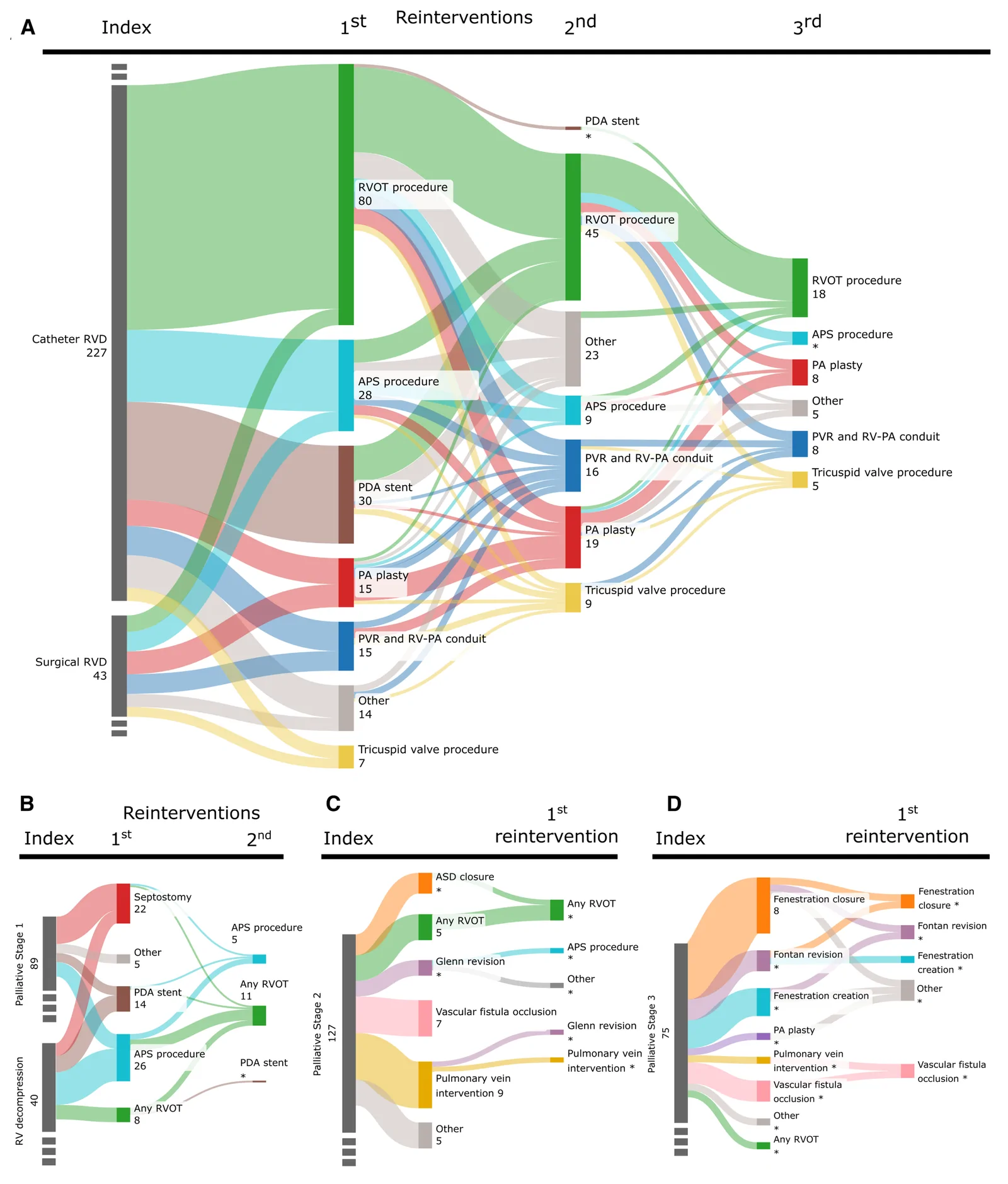
**Sankey diagrams showing reintervention types and sequences. A**, Reinterventions after the initial right ventricular decompression procedure in the biventricular repair (BiV) pathway. **B**, Reinterventions between stages 1 and 2 for functionally single-ventricle (fSV) pathway patients. **C**, Reinterventions after stage 2 in fSV pathway patients. **D**, Reinterventions after stage 3 in fSV pathway patients. Patients with combined procedures are counted multiple times, within each reintervention category. Reinterventions shown are limited to third, second, and first in sequence, respectively, due to small numbers beyond that. APS indicates arteriopulmonary shunt; PA, pulmonary artery; PDA, patent ductus arteriosus; PVR, pulmonary valve replacement; RV, right ventricular; RVD, right ventricular decompression; and RVOT, right ventricular outflow tract.

### Days Spent in Hospital From Birth to 18 Years of Age by Treatment Pathway

In 385 patients with first procedures before March 2017, 14 728 care spells were analyzed. Of these, 2910 (19.8%) were inpatient, 10 852 (73.7%) were outpatient, and 966 (6.6%) were A&E visits without admission.

Median hospital use in the whole cohort was greatest in the first year of life at 35 days (IQR, 20–56), declining to 1 day annually (IQR, 1–2) by age 18 years (Figure 6A and 6B). Before 1 year of age, most days in hospital were inpatient (median, 26; IQR, 16–47). Beyond infancy, inpatient stays were uncommon (most children had no inpatient stays each year), with hospital use largely limited to outpatient visits.

**Figure 6.**
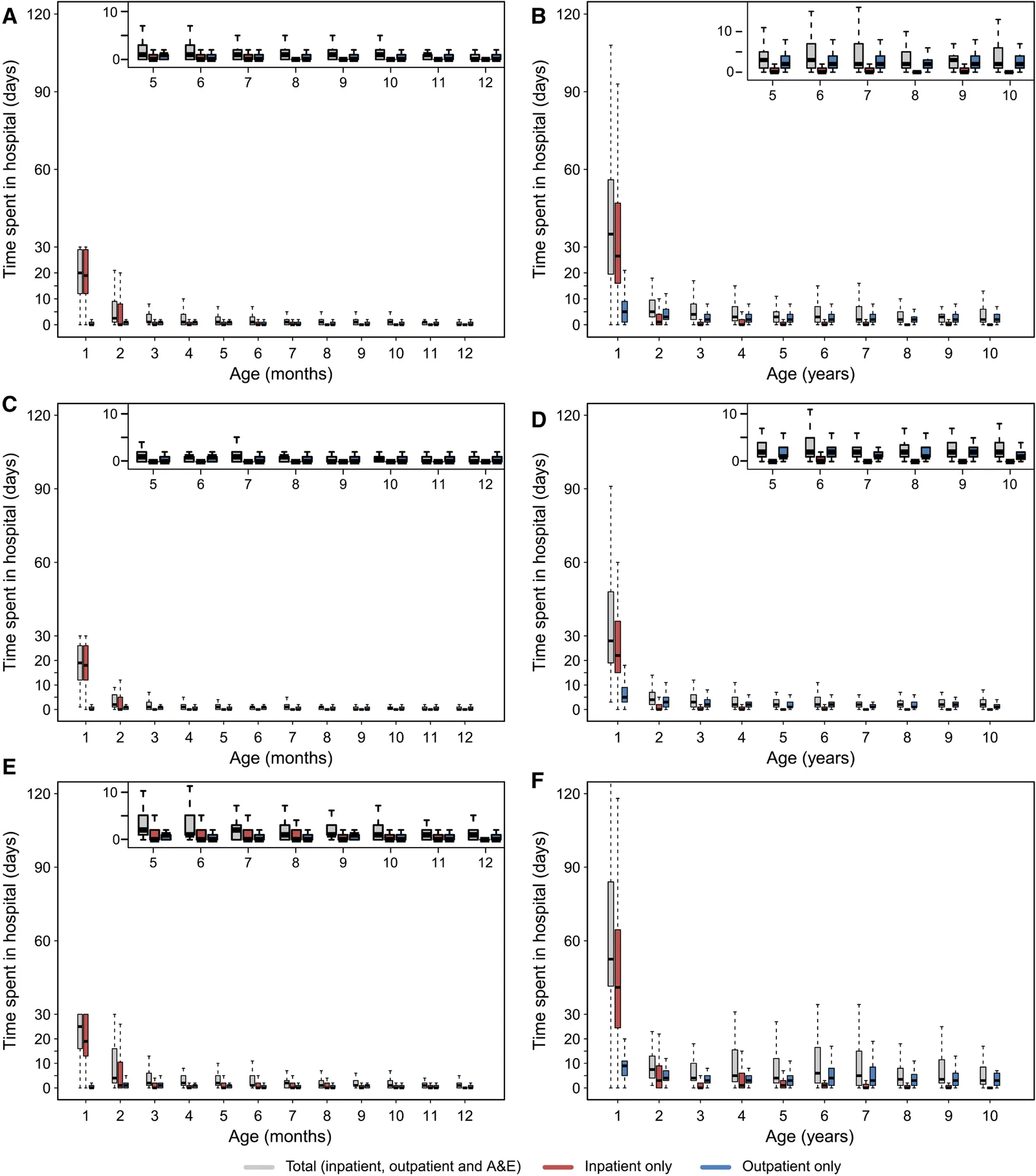
**Days spent in hospital for patients with pulmonary atresia with intact ventricular septum.** Whole cohort (**A**) in the first year of life and (**B**) up to 10 years of age; biventricular pathway patients (**C**) in the first year of life and (**D**) up to 10 years of age; and functionally single-ventricle (fSV) pathway patients (**E**) in the first year of life and (**F**) up to 10 years of age. After the age of 8 years, the median total days spent in hospital remained at 1 day/year. All panels show the median (horizontal black line), interquartile range (colored solid bars), and 1.5× interquartile range (dotted vertical lines). Panel insets are zoomed in beyond month/year 5. Outliers outside these limits are not shown. Numerical values are detailed in Table S10. A&E indicates accident and emergency.

Hospital days in the first year varied by pathway. fSV patients spent more time in hospital (median, 52 days; IQR, 42–82; Figure 6C and 6D) than those on the BiV pathway (median, 28 days; IQR, 19–48; Figure 6E and 6F). Numerical data corresponding to days spent in hospital are shown in Table S10.

## Discussion

In a large national PA-IVS cohort of neonates followed through adolescence, over half achieved BiV repair, comparing favorably with previous reports.^6,15^ The initial interventional approach favored transcatheter RVD, contrasting with earlier multicenter series dominated by APS and ductal stenting, the latter largely in non-BiV candidates. Compared with previous cohorts, outcomes seem more favorable, which coincides with more prevalent transcatheter RVD. However, the exact factors driving these improvements cannot be easily identified in a retrospective, descriptive study (Figure 7A and 7B).^9,15,16^ Palliation with retrograde pulmonary perfusion, particularly APS, continues to carry high mortality, likely reflecting nonmodifiable risks such as RVDCC. A clear disparity persists between the BiV and fSV pathways, with fSV patients having higher early mortality and greater hospital resource use, whereas BiV patients achieve superior survival, shorter hospital stays, but more frequent reinterventions.

**Figure 7.**
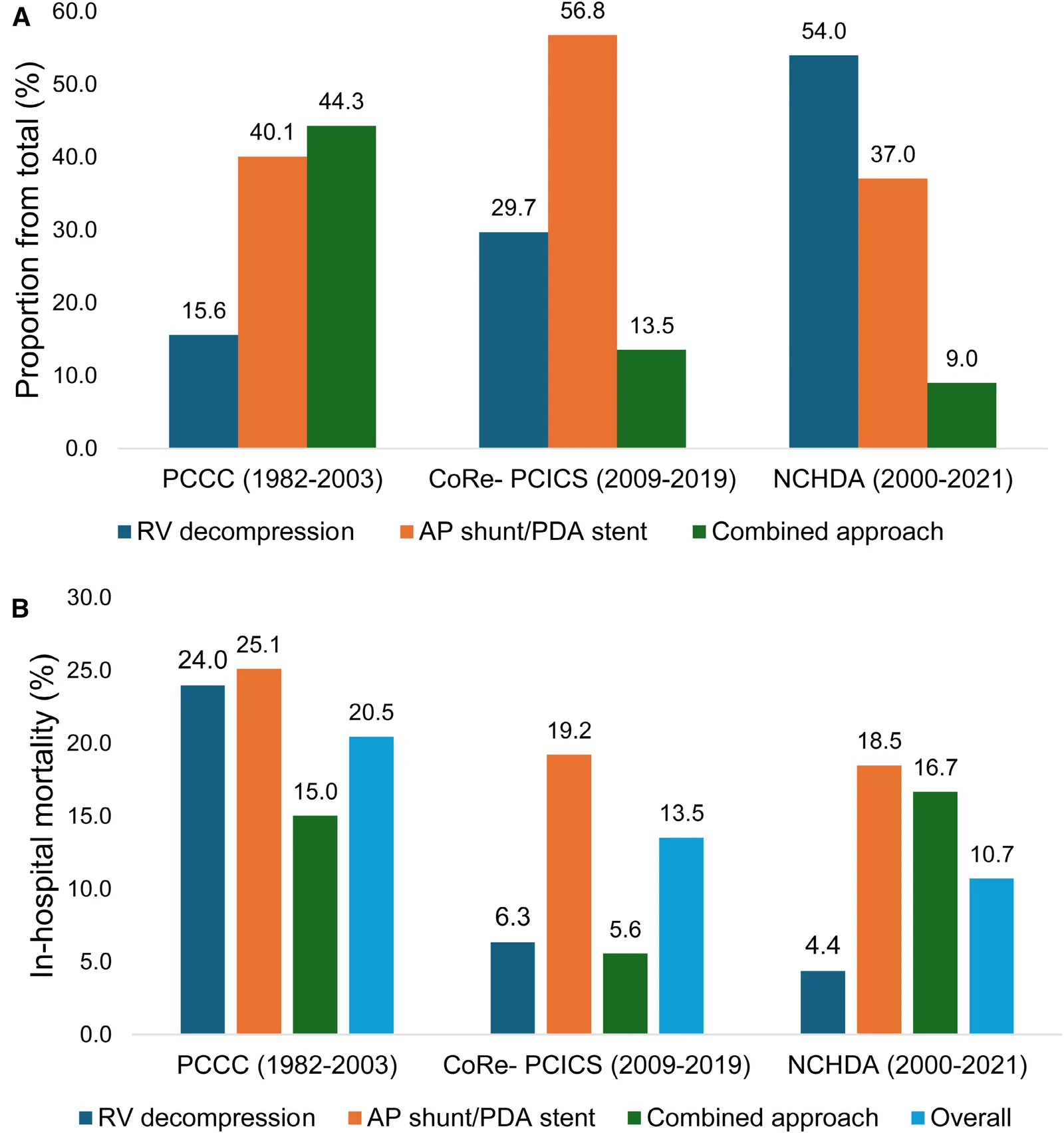
**Comparison between 3 national pulmonary atresia with intact ventricular septum cohorts from 3 eras. A**, Proportion of initial procedure approaches. **B**, In-hospital mortality. Use of right ventricular (RV) decompression increased, with arteriopulmonary (AP) shunt and ductal stenting use decreasing. Overall in-hospital mortality decreased, owing mostly to improved results with RV decompression. CoRE-PCICS indicates Collaborative Research from the Pediatric Cardiac Intensive Care Society Investigators (Iliopoulos et al^10^); NCHDA, National Congenital Heart Disease Audit; PCCC, Pediatric Cardiac Care Consortium (Wright et al^9^); and PDA, patent ductus arteriosus.

PA-IVS management has evolved over the past 2 decades, from early reliance on surgical RVD or fSV palliation to a complex strategy incorporating surgical, transcatheter, and hybrid options, aiming for RV rehabilitation and BiV repair in as many eligible patients as possible.^1^ Three large cohorts of patients treated after 1991, 1997, and 2003, respectively, showed similarly low BiV proportions of <35%, with high mortality before reaching definitive repair, of 30% to 50%.^9,10^ In contrast, the current study shows more favorable contemporary outcomes, with 56.1% of neonates achieving RVD and being committed to BiV and only 15% mortality before definitive BiV or fSV repair. More importantly, there was a considerable increase in 5-year survival after the early 2000s, from 64% to 65% in patients treated before 2003,^9,16^ to 82% to 83% in the more recent cohorts,^10^ consistent with the new UK data presented here. It is difficult to say precisely what led to these improvements, as the relative contributions of evolving intervention strategies, patient selection, institutional practice, and advances in perioperative management cannot be easily disentangled. One aspect that remains constant, and is a reflection of the clinical severity of the PA-IVS spectrum, is a 1-year mortality of 20% to 30%, which has not improved across eras. This is in keeping with the prevalence and mortality seen in cases presenting with RVDCC, an area in which improvements have not been seen.

In BiV-amenable PA-IVS patients, survival after initial RVD is encouragingly high. Transcatheter RVD has remained the preferred strategy in the UK since 2000. Surgical RVD was infrequently used, with higher associated mortality than the transcatheter approach, though this may reflect patient selection for more complex anatomy. Data from the Swedish Registry, albeit in a small group, reported no early deaths with either approach.^3^ In the current study, reintervention profiles were similar between surgical and catheter RVD, particularly regarding APS/ductal stenting, suggesting neither method is superior for securing pulmonary blood flow. Overall, catheter-based RVD strategies have become more widely used in anatomically suitable cases, although superiority over surgical approaches cannot be established from the present observational analysis. Both catheter palliation and surgical RVD carry a substantial reintervention burden in BiV patients, largely anticipated as staged procedures to optimize pulmonary flow and RV rehabilitation. As in other congenital heart diseases, improving survival with minimally invasive strategies may come at the cost of more frequent reinterventions.^17,18^

In the modern UK practice reported here, APS procedures are largely limited to patients continuing on the fSV pathway, with few proceeding to RVD, contrasting with US cohorts.^9,19^ This aligns with UK National Audit findings that APS remains the only congenital heart disease intervention without a reduction in mortality over 2 decades, with PA-IVS showing the highest APS-associated mortality.^20^ Moreover, PA-IVS is the subgroup with the highest mortality associated with APS among all congenital heart diseases. Ductal stenting is emerging as an alternative to APS, with superior procedural outcomes in a recent meta-analysis.^21^ For RVD, catheter-based approaches are increasingly preferred when anatomically feasible, though technical and clinical constraints still limit ductal stenting uptake.^22^ Nevertheless, in this modern UK cohort, almost 20% of antegrade flow augmentation procedures were performed by means of ductal stenting.

In the current cohort, most deaths occurred within the first year, following stage 1 palliation and before definitive commitment to fSV or BiV circulation. These patients likely represent the most severe end of the PA-IVS spectrum, such as those with RVDCC, where therapeutic options remain limited. Historically, survival in this subgroup has shown little improvement, apart from clearer criteria guiding selection for fSV rather than RVD.^1,8,16,19,23^

Data on hospital resource use in PA-IVS are limited. A Utah statewide study reported mean hospital stays of 31 days in the first year and 39 days over 10 years, but included only 33 PA-IVS patients.^24^ Our large cohort provides greater detail. Both fSV and BiV neonates spent most of their first month in hospital, with peak hospital stay occurring in the first year. fSV patients required more hospital days throughout follow-up to age 18 years, reflecting the burden of stage 1 palliation and subsequent palliative operations, as previously described using data from the same UK National Audit.^25^ Differences in hospital resource utilization likely reflect substantial differences in baseline disease severity, anatomic suitability, and patient selection between patients following the fSV versus BiV pathways.

In summary, contemporary UK practice favors catheter-based initial treatment, with APS largely reserved for patients unlikely to achieve BiV repair. This strategy yields outcomes comparable to leading single-center and multicenter series.^6,8,10,26^ The subsequent patient course is shaped less by the initial intervention than by the final pathway achieved. Even so, compared with older large PA-IVS cohorts, a higher proportion of patients now reach BiV repair.^9,15^ Future large studies with more granular clinical and anatomic data are needed to better define evolving patient selection and staged decision-making.

### Limitations

This retrospective analysis relied on linked national registries, without granular data on anatomic variants, imaging, coronary lesions, or RVDCC status, nor on clinical decision-making for initial or subsequent interventions. Comparisons with prior large cohorts allow inference of practice-related associations with outcomes, but causality cannot be established. Data driving the initial pathway choices, especially between BiV and fSV management, are also not documented, so the inherent selection bias remains, especially regarding the baseline anatomic differences between BiV and fSV patients.

Linked data sets varied in start dates and variable availability, restricting analyses to subsets with complete data. Patients without any cardiac procedure could not be identified, so deaths without intervention were not captured, likely representing the most severe cases. When procedures occurred in close succession (eg, RVD and stage 1 palliation), the initial procedure was defined by first occurrence, as the planned/unplanned status of the second could not be determined. Thus, using the definitions used by other groups previously, the prevalence of RVD combined with APS/ductal stenting could be underestimated. These are still reported in the reinterventions section as a descriptive analysis. Patients with one-and-a-half-ventricle circulation repair were few and were included within the fSV group given the physiological similarity.

Given the descriptive nature of this study, any observed differences between treatment pathways should be interpreted with caution, as they likely reflect the sum of all patient clinical characteristics and complex pathway choices, rather than the impact of a single intervention.

### Conclusions

In this contemporary national cohort of PA-IVS, transcatheter RVD has become the predominant initial strategy and is associated with lower early mortality compared with historical series. More than half of patients achieved biventricular repair, a proportion higher than in older cohorts, with national survival into adolescence that compares favorably with the best single-center reports.

Even with improved survival and hospital resource utilization in the BiV group, the burden of reintervention remains substantial, reflecting the staged nature of RV rehabilitation. In contrast, patients managed along the fSV pathway continue to experience higher early mortality and greater hospital use, underscoring the persistent disparity between these pathways.

Despite better BiV patient selection and safer transcatheter RVD, there has been a lack of improvement in practice for the severe end of the PA-IVS spectrum, with early mortality remaining high. This likely reflects the nonmodifiable nature of some of the risk factors. Identifying solutions to these persistent gaps in practice improvement should be the focus of future research, alongside implementing more widespread use of transcatheter interventions in PA-IVS management.

A modern PA-IVS management strategy should encourage transcatheter interventions such as RVD and ductal stenting, rather than APS, along with careful BiV pathway patient selection, taking into account nonmodifiable risk factors and flexible transition between pathways when indicated.

## Article Information

### Disclosures

The authors have no disclosures relevant to this manuscript. The views expressed are those of the authors and not necessarily those of the National Health Service, the National Institute for Health and Care Research, or the Department of Health and Social Care. Syntax, language, and concise writing style were edited using a large language model (Copilot, Microsoft Corporation), and the outputs were checked by the authors for accuracy against the intended original text.

### Supplemental Material

Supplementary Methods

Tables S1–S10

Figure S1

Reference 27

## Supplementary Material
