# Supplementary material for: Pulmonary Atresia With Intact Septum Survival, Reintervention, and Days in Hospital Through Childhood: Contemporary Outcomes From a National Registry
Source: Circ Cardiovasc Interv. 2026 Aug 14;19(10):e016435. doi: 10.1161/CIRCINTERVENTIONS.125.016435 (PMC13674602; doi:10.1161/CIRCINTERVENTIONS.125.016435)
Supplement: Supplementary file 1 [file hcv-19-e016435-s001.pdf]

SUPPLEMENTAL MATERIAL

Dorobantu et al. Pulmonary atresia with intact septum survival, reintervention and days in hospital through childhood: contemporary outcomes from a national registry

Supplementary material table of contents

1. **Patient selection and statistical methodology**
    a. **Patient selection methodology**
    b. **Collected data and outcomes**
    c. **Figure S1:** Linked dataset sources and timeframes
2. **Diagnosis group and treatment pathway classification**
    a. **Table S1**. Pulmonary atresia (PA) diagnosis, procedure codes
    b. **Table S2**. Other CHD exclusion diagnostic, procedure codes
    c. **Table S3**. PA-VSD classification codes
    d. **Table S4**. PA pathway procedure codes
    e. **Table S5.** PA-IVS treatment pathway definitions
3. **Centre variability and early outcomes at first procedure**
    a. **Table S6.** First procedure centre variability
    b. **Table S7.** Early outcomes at the first cardiac procedure
4. **Late outcomes, hospital utilization and associated risk factors**
    a. **Table S8:** Late reintervention probability in biventricular repair group by type
    b. **Table S9.** Reintervention type and timing after RV decompression in biventricular repair group
    c. **Table S10.** Days spent in hospital by year of life and treatment pathway

1. Patient Selection methodology

We selected patients born between 1 April 2000 and 31 March 2017, having a pulmonary atresia with intact septum (PA-IVS) diagnosis, with at least one cardiac procedure performed in England or Wales to guarantee a complete intervention history using the LAUNCHES (Linking Audit and National datasets in Congenital Heart Services for Quality Improvement) dataset. Patients with PA and ventricular septal defect (PA-VSD), including double outlet right ventricle (DORV) or tetralogy of Fallot (ToF) variants or with major aorto-pulmonary collateral arteries (MAPCA) were excluded. Patients born before April 2000 were excluded to ensure complete procedural history. Patients from overseas, Scotland and Northern Ireland were excluded as life status data from the Office of National Statistics does not cover these regions, only England and Wales. This is shown in **Figure S1,** below.

PA diagnosis identification, exclusion of other complex CHD, exclusion of PA-VSD variants, procedure classification automatic steps are described below in the section **"Diagnosis group and treatment pathway classification".**

Collected data and outcomes

All clinical data were organised in "care spells", which can contain procedures, inpatient or intensive care stays, outpatient or emergency room (A&E) visits, in any combination, at no more than one day apart.[11] For each linked dataset, the available data covers different time periods and outcomes, as described in Figure S1.

Data from the following national United Kingdom (UK) sources were collected and linked:

1. National Congenital Heart Disease Audit (NCHDA) as the primary dataset: this contains all records for cardiac procedures done in patients with CHD between April 2000 to March 2021.
2. Office of National Statistics (ONS): provided national death status ascertained for August 2023

3. Hospital episode statistics (HES) dataset: contains inpatient HES data for between April 2000 to March 2018, outpatient for between April 2003 to March 2018 and Accidents & Emergencies (A&E) for between April 2007 to March 2018.
4. Paediatric Intensive Care Audit Network (PICANet): Paediatric Intensive Care Unit data from April 2002 to March 2017.

Linkage methodology and an overview of the dataset were described elsewhere.[11] Hospital episode statistics (HES) and PICANet are only available (linked to the primary dataset) for those who had cardiac intervention records in NCHDA before March 2017.

The following data were collected:

Patient related data: sex, ethnicity, area of residence deprivation index (IMD), preterm birth, antenatal diagnosis, and congenital comorbidity.

Procedure related data: age at procedure, weight at procedure, acquired comorbidity (the presence of an acquired complication related to CHD e.g. necrotising enterocolitis, renal failure), additional cardiac risk factors (associated cardiac conditions or complications not consisting of other CHD, such as pulmonary hypertension, cardiac dysfunction, cardiomyopathy), severity of illness marker (such as circulatory support, shock or acidosis),[12] cardiac procedure type, procedure era and centre. These were previously defined as part of the Pediatric Cardiac Surgery (PRAiS) model.[12]

Hospital resources utilization: hospital length of stay (LoS) and ICU LoS for the first procedure spell, and from birth to end of follow-up: any and cardiac-related inpatient and ICU stays and outpatient or A&E department visits;

Mortality and reintervention: in relation to the first procedure or birth (clearly stated); early mortality and early cardiac reintervention (30 day and in-hospital), mid (1 and 5 years) and late (maximum follow-up) mortality and reintervention (any cardiac, surgical, transcatheter, and by anatomic subtype).

Diagnosis group and treatment pathway classification

The automatic identification of PA-IVS patients using the LAUNCHES dataset followed a multiple step process.

Step 1: Identifying patients with pulmonary atresia (PA)

We identified all patients having a PA diagnosis code, a diagnosis code suggesting presence of PA or a procedure code related to PA repair with or without a ventricular septal defect, as detailed **Table S1**.

Step 2: Exclude patients with more complex/other significant CHD

We excluded patients who have another more complex CHD, based on the codes in **Supplementary Table 2**, as well as those identified as having hypoplastic left heart syndrome (HLHS), functional univentricular heart (FUH), or transposition of the great artery (TGA) in a previous analysis based on the CHAMPION study classification rules.[27] Also excluded were patients with non-contributory procedure records (non-treatment cardiac procedure entered in NCHDA).

Step 3: Classify in PA-IVS and PA-VSD

Patients with diagnostic codes indicative of PA-IVS, that is *010107. Pulmonary atresia + intact ventricular septum, 072100. Intact ventricular septum* or *060101. Tricuspid atresia* were classified as PA-IVS.

Patients with PA-VSD including double outlet right ventricle (DORV) or tetralogy of Fallot (ToF) with major arterio-pulmonary collateral arteries (MAPCA) were identified to be excluded based on the diagnostic and procedure codes in **Table S3**.

Patients with neither definite PA-IVS or PA-VSD codes are classified as PA-IVS and flagged to centres for reconciliation of diagnosis.

Step 4: Identify and classify PA procedures and pathways

The individual types of pathway procedures are “Initial palliative procedure”, “Palliative Stage 2” (Glenn operation type), “Palliative Stage 3” (Fontan operation type) and “RV decompression procedures”. These are identified by procedure codes listed in **Table S4.** The two defined treatment pathways were “single ventricle” and “biventricular”.

Initial palliation and RV decompression procedures can occur on both single ventricle and biventricular pathways. Once a Palliative Stage 2 or 3 occurs, the patient is classified as “single ventricle pathway”. If the only pathway procedure that occurs is RV decompression, with no palliation stage 2 or 3, the patient is classified as “biventricular” pathway.

If multiple types of pathway procedure happened in the same record, then we mark the individual procedure as the most complex one, i.e., the hierarchy/complexity order is palliative procedures in these groups first stage three, then stage two including in this case stage 1.5 ventricle repairs, then reparative procedure for Pulmonary atresia diagnosis, then stage one.

For any of the defined pathway procedures, i.e., palliative procedures (stages one two or three) and RV decompression, only the first occurrence will be the pathway procedure of that type. Other subsequent occurrence of these individual procedures will be identified as reinterventions and off pathway. Patients can have up to 4 pathway procedures in whole history. Of note, additional reparative or stage one procedure(s) after final expected reparative procedure will be identified as additional or off pathway.

Patients who have a procedure before the first pathway procedure are considered to have had a ‘pre-pathway procedure’. Patients who have no pathway procedures are described. Additional off pathway procedures (reinterventions) are described by the stage at which they occur.

Figure S1. Data sources and their time-frames for data collection for linked datasets within the LAUNCHES project.

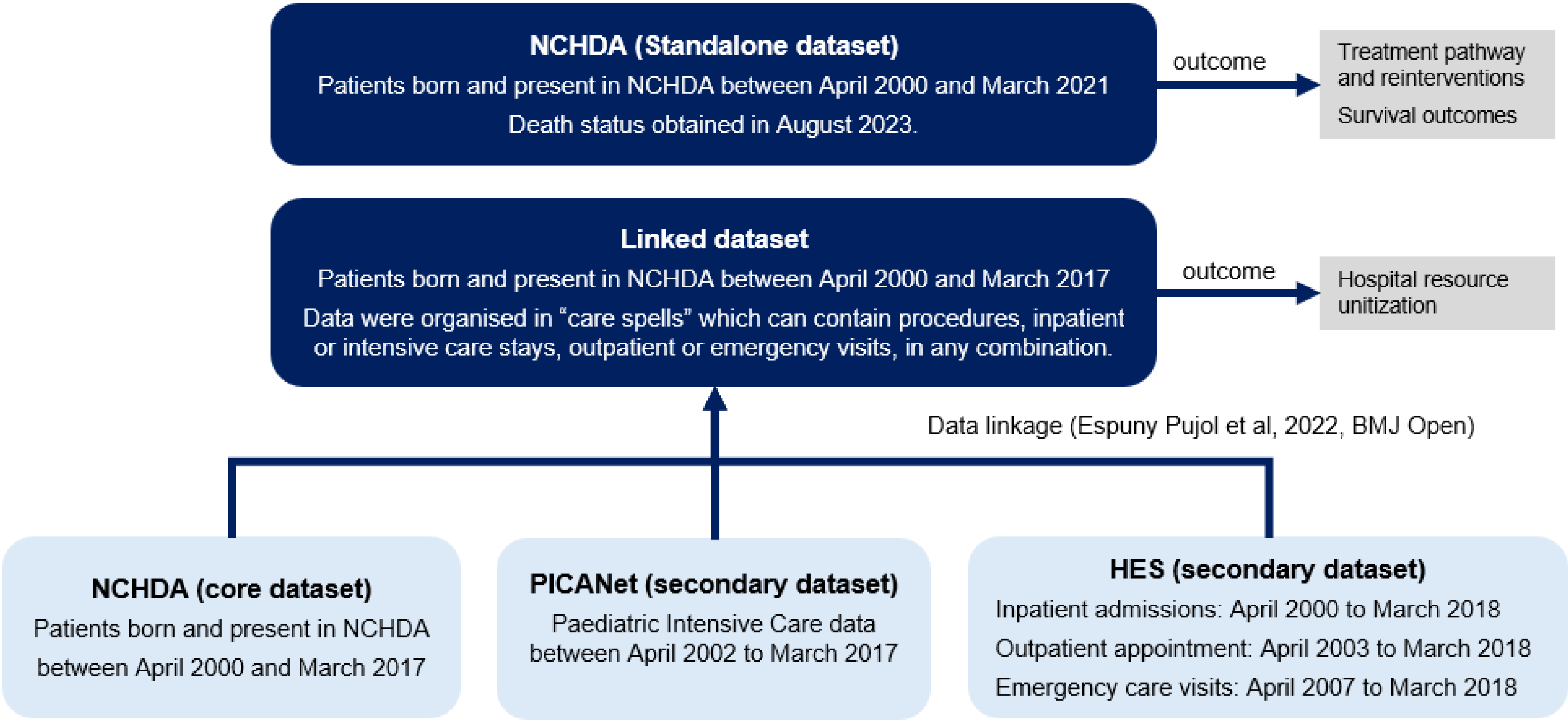

Table S1. Diagnosis and procedure codes used to identify patients with pulmonary atresia, with intact septum or ventricular septal defect

**Main diagnosis codes for classification of pulmonary atresia**
010107. Pulmonary atresia + intact ventricular septum
010106. Pulmonary atresia + VSD (including Fallot type)
010125. Pulmonary atresia +VSD+systemic-to-pulmonary collateral artery(ies) (MAPCA(s))
090908. Pulmonary artery from ascending aorta (hemitruncus)
010126.Tetralogy of Fallot with pulmonary atresia.
010157.Tetralogy of Fallot with pulmonary atresia and systemic-to-pulmonary collateral artery(ies) (MAPCA(s)).
**Diagnosis codes suggestive of pulmonary atresia**
090511. Pulmonary atresia
090512. Pulmonary atresia: imperforate valve
090726. Solitary arterial trunk (absent intrapericardial pulmonary arteries)
090908 Pulmonary artery from descending aorta
091010. Discontinuous (non-confluent) pulmonary arteries
090516.Congenital pulmonary atresia.
090705. Absent or atretic pulmonary trunk (main pulmonary artery).
090911. Pulmonary artery from arterial duct.
091030.Congenitally discontinuous. non-confluent right and left pulmonary arteries.
091075.Absent or atretic right pulmonary artery.
091077.Absent or atretic left pulmonary artery.
**Definite procedure codes for pulmonary atresia repair**
122801. Pulmonary atresia & ventricular septal defect (VSD) (including Fallot-type) repair,
122811. Pulmonary atresia, ventricular septal defect (VSD) & systemic-to-pulmonary collateral artery(ies) (MAPCA(s)) repair,
**Procedure codes very strongly linked to pulmonary atresia diagnosis**
121309. Pulmonary valvar transluminal perforation & dilation
122500 MAPCA Unifocalisation
121430 Pulmonary artery from aorta (hemitrunc) repair

Table S2. Exclusion Diagnostic codes

Diagnosis codes
010109. Hypoplastic left heart syndrome
060201. Mitral atresia
091503. Aortic atresia
010114. Double inlet AV connection (double inlet ventricle)
010122. Functionally univentricular heart
010124. Double outlet right ventricle: with intact ventricular septum
010403. Double inlet RV
010404. Double inlet LV
020305. Solitary ventricle of indeterminate morphology
070841. Functionally univentricular heart
070842. Functionally univentricular heart
090101. Common arterial trunk (truncus arteriosus)
090200. Truncal valvar abnormality
090203. Truncal valvar regurgitation
092931. Interrupted aortic arch
010103. Congenitally corrected transposition of great arteries (discordant AV & VA connections)
010116. Partially anomalous pulmonary venous connections: Scimitar syndrome
010133. Shone's syndrome: left heart obstruction at multiple sites,
010503. Double outlet left ventricle
050201. Cor triatriatum (divided left atrium)
090401. Aortopulmonary window
091600. Supravalvar aortic stenosis
094101. Anomalous origin of coronary artery from pulmonary artery
060134. Ebstein's malformation of tricuspid valve
050601. Common atrium (virtual absence of atrial septum)
060501. AVSD AV valvar abnormality
060506. AVSD AV valvar regurgitation
060600. Atrioventricular septal defect
060601. AVSD: isolated atrial component (primum ASD)(partial)
060608. AVSD: isolated ventricular component
060609. AVSD: atrial & ventricular components with common AV orifice (complete)
060610. AVSD: atrial & (restrictive) ventricular components + separate AV valves ('intermediate')
010120. AV septal defect and Tetralogy of Fallot
060726. AVSD with ventricular imbalance
010117. Double outlet right ventricle: Fallot type (subaortic or doubly committed VSD & pulmonary stenosis)
091501. Aortic valvar stenosis: congenital
091513. Aortic valvar stenosis
091592. Aortic stenosis
050202. Supravalvar mitral ring
060207. Mitral valvar stenosis: congenital
060213. Mitral subvalvar stenosis
060256. Parachute malformation of mitral valve
060292. Mitral stenosis
060293. Mitral valve stenosis
040600. Totally anomalous pulmonary venous connection: supracardiac

040805. Totally anomalous pulmonary venous connection
040806. Obstructed pulmonary venous connection(s)
040810. Totally anomalous pulmonary venous connection: intracardiac
040820. Totally anomalous pulmonary venous connection: infracardiac
040830. Totally anomalous pulmonary venous connection: mixed
030104. Right isomerism ('asplenia')
030105. Left isomerism ('polysplenia')
010118. Double outlet right ventricle: transposition type (subpulmonary VSD)
010501. Discordant VA connections (TGA)
010102. Transposition of great arteries (concordant AV & discordant VA connections) & IVS
070700. Left ventricular hypoplasia
010309 AV and or/VA connections abnormal
090525 Absent pulmonary valve syndrome
091506. Aortic valvar atresia.
010405.Double inlet to solitary ventricle of indeterminate morphology.
020303.Crisscross heart (twisted atrioventricular connections).
060311.Congenital anomaly of right-sided atrioventricular valve in double inlet ventricle.
060411.Congenital anomaly of left-sided atrioventricular valve in double inlet ventricle.
090111. Common arterial trunk (truncus arteriosus) with aortic dominance and one pulmonary artery absent from trunk. isolated pulmonary artery.
090112. Common arterial trunk (truncus arteriosus) with pulmonary dominance and aortic arch obstruction.
090114. Common arterial trunk (truncus arteriosus) with aortic dominance and both pulmonary arteries arising from trunk.
090115. Common arterial trunk (truncus arteriosus) with aortic dominance (no aortic arch obstruction).
090118. Common arterial trunk (truncus arteriosus) with pulmonary dominance and interrupted aortic arch.
090119. Common arterial trunk (truncus arteriosus) with pulmonary dominance and aortic coarctation.
090201.Dysplasia of truncal valve.
090218.Congenital truncal valvar stenosis.
090219.Congenital truncal valvar regurgitation.
010110.Transposition of the great arteries with concordant atrioventricular connections and ventricular septal defect.
092932. Interrupted aortic arch distal to subclavian artery. type A.
092933. Interrupted aortic arch between subclavian & common carotid arteries. type B.
092934. Interrupted aortic arch between carotid arteries. type C.
150503. Pulmonary vein obstruction
020101.Extrathoracic heart (ectopia cordis).
040802.Congenital atresia of pulmonary vein(s).
040804.Congenital anomaly of pulmonary vein(s).
040831.Congenital pulmonary venous stenosis and-or hypoplasia.
090407.Congenital aortopulmonary window.
091618. Congenital supravalvar aortic stenosis.
093134.Vascular ring of left aortic arch and right arterial duct or ligament.
093135.Vascular ring of right aortic arch and left arterial duct or ligament.
094103. Anomalous origin of left coronary artery from pulmonary artery (ALCAPA).
094221.Anomalous aortic origin of coronary artery with ventriculo-arterial concordance.
060598. Deficient mural-lateral leaflet of left ventricular component of common atrioventricular valve (left atrioventricular vale)
050603.Common atrium with common atrioventricular junction.
060514.Atypical common atrioventricular valve.

060525. Double orifice of left ventricular component of common atrioventricular valve (left atrioventricular valve).
060560.Common atrioventricular valvar regurgitation.
060571.Atypical right ventricular component of common atrioventricular valve (right atrioventricular valve).
060572.Atypical left ventricular component of common atrioventricular valve (left atrioventricular valve).
060705. Atrioventricular septal defect (AVSD) with ventricular imbalance with dominant right ventricle and hypoplastic left ventricle.
060706. Atrioventricular septal defect (AVSD) with ventricular imbalance with dominant left ventricle and hypoplastic right ventricle.
060727. Atrioventricular septal defect (AVSD) with balanced ventricles.
060728.Common atrioventricular junction with spontaneous fibrous closure of atrioventricular septal defect (AVSD).
060736. Common atrioventricular valve with unbalanced commitment of valve to ventricles.
060737.Common atrioventricular valve with unbalanced commitment of valve to right ventricle.
060738.Common atrioventricular valve with unbalanced commitment of valve to left ventricle.
060202. Mitral atresia with imperforate mitral valve.
060203.Dysplasia of mitral valve.
060204. Mitral annular hypoplasia.
060205. Overriding mitral valve.
060211.Congenital anomaly of mitral valve.
060221.Congenital anomaly of mitral subvalvar apparatus.
060222. Congenital mitral subvalvar stenosis.
060226. Mitral atresia with absent valvar annulus (connection-junction).
060272. Congenital mitral valvar prolapse.
040807. Anomalous pulmonary venous connection.
091517. Aortic 'annular' hypoplasia.
092944.Descending thoracic or abdominal aortic coarctation.

Procedure exclusion codes (linked to other defects)

122911 Rastelli
122621 Absent pulmonary valve syndrome repair
122745 REV procedure
121100 Common arterial trunk repair
121201 Aortopulmonary window repair
121402 Pulmonary trunk band
121312 Pulmonary valvectomy

Table S3. Diagnosis and procedure codes associated with pulmonary atresia and ventricular septal defect

**Diagnosis codes**
010101. Tetralogy of Fallot
010106. Pulmonary atresia + VSD (including Fallot type)
010125. Pulmonary atresia + VSD + systemic-to-pulmonary collateral artery(ies) (MAPCA(s))
071000. VSD
071001. Perimembranous VSD
071012. VSD + malaligned outlet septum
071101. Muscular VSD
071200. Subarterial VSD
071201. Doubly committed subarterial VSD
071402. Communication between left ventricle + right atrium (Gerbode defect)
071405. Inlet VSD
071501. Tiny VSD (Maladie de Roger)
071504. Multiple VSDs
071505. Single VSD
010119 DORV with uncommitted VSD
010104 Double outlet right ventricle
090726. Solitary arterial trunk (absent intrapericardial pulmonary arteries)
090801. Major systemic-to-pulmonary collateral artery(ies) (MAPCA(s))
092025. Systemic-to-pulmonary collateral arter(ies) (MAPCA(s)) stenosis(es)
071002.Inlet perimembranous ventricular septal defect (VSD) without atrioventricular malalignment without a common atrioventricular junction.
071004. Outlet perimembranous ventricular septal defect (VSD) with anteriorly malaligned of outlet septum.
071017.Outlet ventricular septal defect (VSD) with anteriorly malaligned outlet septum.
071018.Outlet ventricular septal defect (VSD) with posteriorly malaligned outlet septum.
071019. Outlet perimembranous ventricular septal defect (VSD) with posteriorly malaligned outlet septum.
071102.Inlet muscular ventricular septal defect (VSD).
071103.Trabecular muscular ventricular septal defect (VSD) apical.
071104.Trabecular muscular ventricular septal defect (VSD) midseptal.
071105.Trabecular muscular ventricular septal defect (VSD)s multiple (Swiss cheese septum).
071106. Outlet muscular ventricular septal defect (VSD) without malalignment.
071107.Trabecular muscular ventricular septal defect (VSD) anterosuperior.
071112.Trabecular muscular ventricular septal defect (VSD) postero-inferior.
071115. Outlet muscular ventricular septal defect (VSD) with anteriorly malaligned outlet septum.
071116. Outlet muscular ventricular septal defect (VSD) with posteriorly malaligned outlet septum.
071202. Doubly committed juxta-arterial ventricular septal defect (VSD) without malalignment and with muscular postero-inferior rim.
071203. Doubly committed juxta-arterial ventricular septal defect (VSD) without malalignment and with perimembranous extension.
071205. Doubly committed juxta-arterial ventricular septal defect (VSD) with anteriorly malaligned fibrous outlet septum and perimembranous extension.

071206. Doubly committed juxta-arterial ventricular septal defect (VSD) with posteriorly malaligned fibrous outlet septum and perimembranous extension.
071207. Doubly committed juxta-arterial ventricular septal defect (VSD) with anteriorly malaligned fibrous outlet septum and muscular postero-inferior rim.
071208. Doubly committed juxta-arterial ventricular septal defect (VSD) with posteriorly malaligned outlet septum and muscular postero-inferior rim.
071209.Outlet ventricular septal defect (VSD) without malalignment.
071212.Doubly committed juxta-arterial ventricular septal defect (VSD) with anteriorly malaligned fibrous outlet septum.
071213.Doubly committed juxta-arterial ventricular septal defect (VSD) with posteriorly malaligned fibrous outlet septum.
071406.Inlet perimembranous ventricular septal defect (VSD) with atrioventricular septal malalignment and without common atrioventricular junction.

Procedure codes

122801. Pulmonary atresia & ventricular septal defect (VSD) (including Fallot-type) repair,
122811. Pulmonary atresia, ventricular septal defect (VSD) & systemic-to-pulmonary collateral artery(ies) (MAPCA(s)) repair,
122500. Systemic-to-pulmonary collateral artery (ies) (MAPCA(s)) unifocalisation procedure,
121430. Pulmonary artery origin from ascending aorta (hemitruncus) repair,
010117. Double outlet right ventricle: Fallot type (subaortic or doubly committed VSD & pulmonary stenosis)
120801. VSD closure
120802. VSD closure by direct suture
120803. VSD closure using patch
120828. VSD closure with prosthesis - intraoperatively
122601. Tetralogy of Fallot repair,
122613. Tetralogy of Fallot repair with transannular patch,
122620. Tetralogy of Fallot repair without transannular patch,
122518. Systemic-to-pulmonary collateral artery(ies) (MAPCA(s)) occlusion,
122519. Transluminal procedure to systemic-to-pulmonary collateral artery (MAPCA(s)),
122562. Stent placement in systemic-to-pulmonary collateral artery (MAPCA(s)),
122565. Transluminal occlusion of systemic-to-pulmonary collateral artery(ies) (MAPCA(s)) with coil-device,
122572. Balloon dilation of systemic-to-pulmonary collateral artery(ies) (MAPCA(s)),
122567. Unilateral Systemic-to-pulmonary collateral artery(ies) (MAPCA(s)) unifocalisation procedure.
122587. Bilateral systemic-to-pulmonary collateral artery(ies) (MAPCA(s)) unifocalisation procedure: complete with all usable collaterals incorporated.
122588. Bilateral systemic-to-pulmonary collateral artery(ies) (MAPCA(s)) unifocalisation procedure: incomplete with not all usable collaterals incorporated.
122821. Pulmonary atresia. ventricular septal defect (VSD) & systemic-to-pulmonary collateral artery(ies) (MAPCA(s)) repair: status post complete unifocalisation with all usable collaterals incorporated.
122822. Pulmonary atresia. ventricular septal defect (VSD) & systemic-to-pulmonary collateral artery(ies) (MAPCA(s)) repair: status post incomplete unifocalisation with not all usable collaterals incorporated.

Table S4. Pulmonary atresia treatment pathway classification codes

**Right ventricular decompression procedure or repair procedure**
122801. Pulmonary atresia & ventricular septal defect (VSD) (including Fallot-type) repair,
122811. Pulmonary atresia, ventricular septal defect (VSD) & systemic-to-pulmonary collateral artery(ies) (MAPCA(s)) repair,
122821. Pulmonary atresia. ventricular septal defect (VSD) & systemic-to-pulmonary collateral artery(ies) (MAPCA(s)) repair: status post complete unifocalisation with all usable collaterals incorporated.
122822. Pulmonary atresia. ventricular septal defect (VSD) & systemic-to-pulmonary collateral artery(ies) (MAPCA(s)) repair: status post incomplete unifocalisation with not all usable collaterals incorporated.
122500. Systemic-to-pulmonary collateral artery (ies) (MAPCA(s)) unifocalisation procedure,
121430. Pulmonary artery origin from ascending aorta (hemitruncus) repair,
122920 Double outlet right ventricle repair
122567. Unilateral Systemic-to-pulmonary collateral artery(ies) (MAPCA(s)) unifocalisation procedure.
122587. Bilateral systemic-to-pulmonary collateral artery(ies) (MAPCA(s)) unifocalisation procedure: complete with all usable collaterals incorporated.
122588. Bilateral systemic-to-pulmonary collateral artery(ies) (MAPCA(s)) unifocalisation procedure: incomplete with not all usable collaterals incorporated.
121300. Pulmonary valvar procedure,
121302. Pulmonary valvotomy: open,
121321. Pulmonary valvar replacement (not conduit),
121322. Pulmonary valvar replacement using homograft,
123601. Right ventricle to pulmonary arterial tree conduit construction,
120641 Right ventricular outflow tract obstruction relief
120600 Right ventricular outflow tract procedure
120643 RVPulmonary Atresia Sano type
121420. Pulmonary arterioplasty/ reconstruction,
121401. Pulmonary trunk arterioplasty
121421. Pulmonary arterioplasty / reconstruction
121422. Pulmonary arterioplasty peripheral
120612. Right ventricle to pulmonary artery valveless conduit construction.
120647. Right ventricular outflow tract reconstruction: transannular patch & constructed monocuspid valve.
121305. Balloon dilatation of the pulmonary valve
120605. Balloon dilation of the RVOT
123600. Conduit construction
123640. Procedure involving a conduit
**Initial palliation**
121014: Stent placement in arterial duct (PDA)
123103: Modified R Blalock interposition shunt

123104: Modified L Blalock interposition shunt

123106: Central systemic-Pulmonary Atresia interposition shunt

123130: Systemic-to-pulmonary arterial shunt procedure

123146: Modified Blalock interposition shunt

Palliative stage 2 (Glenn operation)

123111: Bidirectional superior cavopulmonary (Glenn) anastomosis

123115: Hemi-Fontan procedure

123144: Bilateral bidirectional superior cavopulmonary (Glenn) anastomoses

123145: Unidirectional superior cavopulmonary (Glenn) anastomosis

123144: Bilateral bidirectional superior cavopulmonary (Glenn) anastomoses

123145: Unidirectional superior cavopulmonary (Glenn) anastomosis

123172: Superior caval vein to pulmonary artery anastomosis.

1.5 ventricle repair

120619. 1.5 ventricle repair: superior cavopulmonary (Glenn) anastomosis + right ventricular outflow tract reconstruction.

Palliative stage 3 (Fontan operation)

123001: Fontan type procedure

123005: Total cavopulmonary connection (TCPC) using extracardiac inferior caval vein (IVC)-pulmonary artery conduit with fenestration

123006: Total cavopulmonary connection (TCPC) with fenestrated lateral atrial tunnel

123013: Fontan procedure with atrioventricular connection

123028: Fontan-type connection without fenestration

123032: Fontan procedure with direct atriopulmonary anastomosis

123050: Total cavopulmonary connection (TCPC)

123051: Total cavopulmonary connection (TCPC) with lateral atrial tunnel

123054: Total cavopulmonary connection (TCPC) using extracardiac inferior caval vein (IVC)-pulmonary artery conduit

123060: Completion of total cavopulmonary connection (TCPC) using transcatheter inferior to superior caval vein covered stent

123092.Total cavopulmonary connection (TCPC) using intra-extracardiac conduit: fenestrated.

123093.Total cavopulmonary connection (TCPC) using intra-extracardiac conduit: nonfenestrated.

Table S5. Classification of treatment pathways in pulmonary atresia with intact septum patients undergoing treatment

Reparative/bi-ventricle (BiV) pathway	Single ventricle (SV) pathway	Non-committed pathway
Transcatheter or surgical right ventricular decompression (RVD) procedures to secure antegrade blood flow through the right ventricular outflow tract (RVOT) and no palliative stage 2 or 3	Either or both palliative stage 2 and 3 interventions including one-and-a-half ventricle repairs (1.5V) who had a surgical type of reparative procedure and palliative stage 2	A pre-pathway or an initial palliative procedure only, who then died or were lost to follow-up, before committing to a pathway

Definitions:

- ***Pre-pathway:*** atrial septostomy (59%), pulmonary artery (PA) interventions (16%) and other miscellaneous procedures (25%).
- *Initial palliation*: Procedures to secure pulmonary blood flow including systemic to PA shunts and ductal shunts.
- ***Palliative stage 2***: Glenn operation (in isolation), Glenn operation with additional reparative procedures, 1.5V type repair.
- ***Palliative stage 3***: Fontan operation

Table S6. First pathway procedure choice variability by center.

	UK1	UK2	UK3	UK4	UK5	UK6	UK7	UK8	UK9	UK10	UK11	UK12
**First treatment pathway procedure**												
Any RVD	51 (71.8%)	41 (50.6%)	27 (51.9%)	15 (53.6%)	38 (64.4%)	25 (71.4%)	37 (74.0%)	19 (79.2%)	37 (80.4%)	9 (69.2%)	23 (65.7%)	None
Initial palliation	20 (28.2%)	40 (49.4%)	25 (48.1%)	13 (46.4%)	21 (35.6%)	10 (28.6%)	13 (26.0%)	5 (20.8%)	9 (19.6%)	4 (30.8%)	12 (24.3%)	1 (100.0%)
**RVD type**												
Surgical RVD	2 (3.9%)	9 (22.0%)	5 (18.5%)	2 (13.3%)	6 (15.8%)	2 (8.0%)	2 (5.4%)	3 (15.8%)	8 (21.6%)	5 (55.6%)	5 (21.7%)	None
Catheter RVD	49 (96.1%)	32 (78.0%)	22 (81.5%)	13 (86.7%)	32 (84.2%)	23 (92.0%)	35 (94.6%)	16 (84.2%)	29 (78.4%)	4 (44.4%)	18 (78.3%)	None
**Initial palliation type**												
PDA stent	7 (35.0%)	6 (15.0%)	3 (12.0%)	4 (30.8%)	5 (23.8%)	1 (10.0%)	none	4 (80.0%)	1 (11.1%)	none	2 (16.7%)	none
APS	13 (65.0%)	34 (85.0%)	22 (88.0%)	9 (69.2%)	16 (76.2%)	9 (90.0%)	13 (100.0%)	1 (20.0%)	8 (88.9%)	4 (100.0%)	10 (83.3%)	1 (100.0%)

Centers are anonymized and numbered from UK1-UK12. Values are presented as n and % from center total.

Table S7. Early outcomes at the first cardiac procedure in PA-IVS neonates, overall and by first procedure type.

Outcome type	RV decompression as first intervention		Initial palliation as first intervention		Overall cohort at first intervention	
	Size of patient group	Outcome measure	Size of patient group	Outcome measure	Size of patient group	Outcome measure
**Age at procedure (median days (IQR)**	n=287	4 (2-7)	n=152	6 (4, 10)	n=488	5 (3-9)
**30 day mortality, n (%)[a]**	n=284	18 (6.3%)	n=150	31 (20.7%)	n=482	61 (12.7%)
**In-hospital mortality, n (%)**	n=287	17 (5.9%)	n=152	28 (18.4%)	n=488	57 (11.7%)
**Hospital length of stay[b]**						
Total (days)	n=217	19 (12-30)	n=124	16 (10-29)	n=378	18 (11-29)
Pre-procedural	n=217	3 (2-6)	n=124	5 (3-7)	n=378	4 (2-6)
Post- procedural	n=217	16 (8-25)	n=124	11 (7-22)	n=378	14 (8-24)
**ICU length of stay[c]**						
Total (days)	n=150	5 (3-10)	n=102	5 (3-12)	n=281	5 (3-10)
Pre-procedural	n=150	1 (0-2)	n=102	1 (1-3)	n=290	1 (0-2)
Post- procedural	n=150	4 (1-7)	n=102	4 (1-8)	n=290	4 (1-7)

Data are n(%) or median (IQR).

[a] does not include n=3 those censored with less than 30 days after repair

[b] Hospital utilization data were available for n=278 patients who underwent the first cardiac procedure before March 2017.

[c] PICANet data were available in n=290 patients who underwent the first cardiac procedure between April 2002 and March 2017.

Table S8. Late cardiac reinterventions after right ventricular decompression (RVD) by type in biventricular repair pulmonary atresia – intact septum (PA-IVS) patients

	Reintervention after RVD							
	All cardiac re intervention		***PV/RVOT reintervention***		***PDA stent, shunt or septostomy procedure***		***Pulmonary artery reintervention***	
Months from RVD	number at risk	cumulative incidence	number at risk	cumulative incidence	number at risk	cumulative incidence	number at risk	cumulative incidence
0	270		270		270		270	
1	173	29.3% (23.9%-34.8%)	222	10.1% (6.8%-14.1%)	197	18.8% (14.3%-23.7%)	241	1.1% (0.3%-3.0%)
5	136	42.5% (36.4%-48.4%)	175	25.2% (20.1%-30.6%)	187	19.9% (15.4%-25.0%)	228	2.6% (1.2%-5.1%)
12	111	51.1% (44.9%-57.0%)	140	35.8% (30.0%-41.6%)	181	19.9% (15.4%-25.0%)	214	3.9% (2.0%-6.7%)
	All cardiac re intervention		***PV/RVOT reintervention***		***PDA stent, shunt or septostomy procedure***		***Pulmonary artery reintervention***	
Years from RVD	number at risk	cumulative incidence	number at risk	cumulative incidence	number at risk	cumulative incidence	number at risk	cumulative incidence
0	270		270		270		270	
1	111	51.1% (44.9%-57.0%)	140	35.8% (30.0%-41.6%)	181	19.9% (15.4%-25.0%)	214	3.9% (2.0%-6.7%)
3	90	56.5% (50.2%-62.3%)	117	41.2% (35.1%-47.2%)	165	19.9% (15.4%-25.0%)	185	8.4% (5.4%-12.3%)
5	75	58.2% (51.9%-64.0%)	100	43.0% (36.9%-49.1%)	144	20.4% (15.8%-25.5%)	160	9.8% (6.4%-13.9%)
7	54	62.6% (56.1%-68.4%)	81	45.2% (38.9%-51.3%)	126	20.4% (15.8%-25.5%)	138	10.9% (7.3%-15.2%)
9	46	62.6% (56.1%-68.4%)	67	45.8% (39.4%-52.0%)	106	20.4% (15.8%-25.5%)	116	11.4% (7.7%-16.0%)
11	37	64.3% (57.6%-70.2%)	54	48.1% (41.4%-54.5%)	92	20.4% (15.8%-25.5%)	101	12.9% (8.8%-17.8%)
13	30	65.2%	44	49.0%	75	20.4%	81	12.9%

		(58.4%-71.2%)		(42.2%-55.5%)		(15.8%-25.5%)		(8.8%-17.8%)
15	24	67.4% (60.2%-73.6%)	34	51.1% (43.9%-57.8%)	57	20.4% (15.8%-25.5%)	63	14.1% (9.5%-19.5%)
17	14	69.0% (61.3%-75.5%)	18	56.1% (47.4%-64.0%)	38	20.4% (15.8%-25.5%)	44	14.1% (9.5%-19.5%)
19	8	73.9% (64.0%-81.4%)	11	61.0% (50.4%-70.0%)	25	20.4% (15.8%-25.5%)	27	14.1% (9.5%-19.5%)
21	*	NA	*	NA	7	20.4% (15.8%-25.5%)	6	18.0% (10.0%-28.0%)

For each time point, the table contains the number left at risk at that time (did not have a reintervention, did not die and were not lost due to data censoring) and the estimated cumulative incidence functions (CIF) of reintervention with 95% CI.

Table S9. Cardiac reintervention types and timing in PA-IVS patients undergoing biventricular repair, by initial RV decompression strategy.

	**All RV decompression**		**Surgical RV decompression**		**Catheter RV decompression**	
	n (%)	Time of event	n (%)	Time of event	n (%)	Time of event
**Any type of reintervention**	167 (61.9%)	37 (8, 265)	27 (62.8%)	26 (4, 381)	140 (61.7%)	42 (9, 233)
**By reintervention types**						
Surgical	97 (35.9%)	138 (4, 767)	23 (53.5%)	129 (5, 2836)	74 (32.6%)	162 (4, 708)
Catheter	128 (47.4%)	94 (21, 364)	15 (34.9%)	200 (56, 814)	113 (49.8%)	84 (20, 315)
PA & RVOT	133 (49.3%)	137 (36, 395)	22 (51.2%)	184 (24, 1022)	111 (48.9%)	135 (38, 353)
PV/RVOT reintervention	126 (46.7%)	138 (37, 386)	17 (39.5%)	200 (19, 3653)	109 (48.0%)	135 (41, 327)
PA reintervention	31 (11.5%)	466 (227, 1327)	12 (27.9%)	463 (70, 1118)	19 (8.4%)	466 (321, 2268)
PDA stent, shunt or septostomy procedure	54 (20.0%)	10 (4, 18)	7 (16.3%)	4 (2, 16)	47 (20.7%)	10 (4, 17)
PDA stent	31 (11.5%)	15 (9, 22)	none	NA	31 (13.7%)	15 (9, 22)
Shunt	33 (12.2%)	4 (2, 16)	9 (20.9%)	6 (2, 647)	24 (10.6%)	4 (2, 11)
ASD closure	33 (12.2%)	2025 (1204, 3376)	5 (11.6%)	1895 (1421, 4564)	28 (12.3%)	2062 (1142, 3250)
Tricuspid valve procedure	20 (7.4%)	1324 (253, 2447)	5 (11.6%)	1533 (167, 2020)	15 (6.6%)	1288 (300, 2598)

The frequency reflects those having at least one event of the respective type (some might have several, counted once). Time of each reintervention type is calculated using the first instance for each respective type and reported as median (IQR).

Table S10. Days spent in hospital, including inpatient, outpatient and A&E, for the whole cohort of pulmonary atresia with intact septum (PA-IVS) neonates, those undergoing biventricular (BiV) repair and functionally single ventricle (fSV) pathway

Whole cohort of PA-IVS

Monthly interval from birth	Total time in hospital (days)								
	Total (inpatient, outpatient and A&E)			***Inpatient only***			***Outpatient only***		
	Number of patients	median (IQR)	range	Number of patients	median (IQR)	range	Number of patients	median (IQR)	range
1st month	192	20 (12-29)	(0-30)	344	19 (12-29)	(0-30)	291	0 (0-1)	(0-9)
2nd month	168	2 (1-9)	(0-30)	312	0 (0-8)	(0-30)	250	1 (0-1)	(0-8)
5th month	156	1 (0-3)	(0-30)	291	0 (0-1)	(0-30)	234	1 (0-1)	(0-6)
12th month	161	0 (0-1)	(0-30)	281	0 (0-0)	(0-30)	236	0 (0-1)	(0-6)

**Yearly interval from birth**	**Total time in hospital (days)**								
	Total (inpatient, outpatient and A&E)			***Inpatient only***			***Outpatient only***		
	**Number of patients**	**median (IQR)**	**range**	**Number of patients**	**median (IQR)**	**range**	**Number of patients**	**median (IQR)**	**range**
1st year	192	35 (20-56)	(0-365)	344	26 (16-47)	(0-365)	291	5 (1-9)	(0-39)
2nd year	164	5 (3-9)	(0-155)	283	1 (0-4)	(0-260)	234	3 (2-6)	(0-18)
3rd year	171	4 (2-8)	(0-128)	268	0 (0-1)	(0-123)	241	2 (1-4)	(0-59)
4th year	167	3 (2-7)	(0-73)	250	0 (0-2)	(0-58)	239	2 (1-4)	(0-67)
5th year	164	3 (1-5)	(0-82)	227	0 (0-1)	(0-80)	216	2 (1-4)	(0-72)
6th year	156	3 (1-7)	(0-161)	207	0 (0-1)	(0-161)	195	2 (1-4)	(0-54)
8th year	170	2 (1-5)	(0-77)	183	0 (0-0)	(0-76)	173	2 (1-3)	(0-52)
10th year	135	2 (1-6)	(0-45)	145	0 (0-0)	(0-22)	137	2 (1-4)	(0-41)
12th year	110	2 (1-5)	(0-50)	117	0 (0-0)	(0-23)	112	2 (1-4)	(0-32)
14th year	77	3 (1-5)	(0-37)	81	0 (0-1)	(0-33)	79	2 (1-4)	(0-26)
16th year	47	3 (1-6)	(0-27)	51	0 (0-1)	(0-5)	49	2 (1-4)	(0-20)
18th year	15	1 (1-2)	(0-18)	17	0 (0-0)	(0-9)	15	1 (0-2)	(0-16)

Patients underwent functionally single ventricle (fSV) pathway

Monthly interval from birth	Total time in hospital (days)								
	Total (inpatient, outpatient and A&E)			*Inpatient only*			*Outpatient only*		
	Number of patients	median (IQR)	range	Number of patients	median (IQR)	range	Number of patients	median (IQR)	range
1st month	52	25 (16-30)	(0-30)	104	19 (13-30)	(0-30)	82	0 (0-1)	(0-5)
2nd month	54	4 (2-16)	(0-30)	104	1 (0-10)	(0-30)	82	1 (0-2)	(0-8)
5th month	54	2 (1-5)	(0-30)	104	0 (0-2)	(0-30)	85	1 (0-1)	(0-6)
12th month	58	1 (0-2)	(0-30)	104	0 (0-0)	(0-30)	87	0 (0-1)	(0-6)

Yearly interval from birth	Total time in hospital (days)								
	Total (inpatient, outpatient and A&E)			*Inpatient only*			*Outpatient only*		
	Number of patients	median (IQR)	range	Number of patients	median (IQR)	range	Number of patients	median (IQR)	range
1st year	52	52 (42-82)	(0-345)	104	41 (25-64)	(0-342)	82	9 (5-11)	(0-39)
2nd year	60	8 (4-13)	(1-46)	106	3 (0-9)	(0-260)	86	4 (3-7)	(0-18)
3rd year	63	4 (3-10)	(0-128)	100	0 (0-2)	(0-123)	89	3 (2-5)	(0-14)
4th year	64	5 (3-15)	(0-71)	94	1 (0-6)	(0-58)	88	3 (2-5)	(0-27)
5th year	57	4 (2-12)	(0-82)	83	1 (0-3)	(0-80)	77	3 (1-5)	(0-29)
6th year	56	6 (2-15)	(0-161)	77	1 (0-2)	(0-161)	70	4 (1-8)	(0-50)
8th year	62	4 (1-8)	(0-77)	69	0 (0-1)	(0-76)	63	3 (1-6)	(0-30)
10th year	48	3 (2-8)	(0-43)	54	0 (0-0)	(0-14)	48	3 (1-6)	(0-29)
12th year	40	3 (1-7)	(0-50)	44	0 (0-0)	(0-23)	40	2 (1-5)	(0-32)
14th year	27	3 (2-7)	(0-37)	29	0 (0-1)	(0-33)	27	2 (1-5)	(0-26)
16th year	17	2 (1-6)	(0-27)	19	0 (0-0)	(0-5)	17	2 (1-4)	(0-20)
18th year	*	NA	NA	6	0 (0-0)	(0-9)	*	NA	NA

Patients underwent biventricular (BiV) pathway

Monthly interval from birth	Total time in hospital (days)								
	Total (inpatient, outpatient and A&E)			*Inpatient only*			*Outpatient only*		
	Number of patients	median (IQR)	range	Number of patients	median (IQR)	range	Number of patients	median (IQR)	range
1st month	103	19 (12-26)	(1-30)	185	18 (12-26)	(0-30)	152	0 (0-1)	(0-3)
2nd month	93	2 (1-6)	(0-30)	174	0 (0-5)	(0-30)	136	1 (0-1)	(0-7)
5th month	92	1 (0-2)	(0-30)	171	0 (0-0)	(0-30)	134	0 (0-1)	(0-4)
12th month	97	0 (0-1)	(0-30)	168	0 (0-0)	(0-30)	139	0 (0-1)	(0-2)

Yearly interval from birth	Total time in hospital (days)								
	Total (inpatient, outpatient and A&E)			*Inpatient only*			*Outpatient only*		
	patient number	median (IQR)	range	patient number	median (IQR)	range	patient number	median (IQR)	range
1st year	103	28 (19-48)	(3-365)	185	22 (15-36)	(0-365)	152	5 (3-9)	(0-36)
2nd year	98	4 (2-7)	(0-155)	170	0 (0-2)	(0-143)	140	3 (1-5)	(0-14)
3rd year	103	3 (1-6)	(0-87)	162	0 (0-1)	(0-74)	145	2 (1-4)	(0-59)
4th year	99	2 (1-5)	(0-73)	151	0 (0-1)	(0-22)	146	2 (1-3)	(0-67)
5th year	103	2 (1-4)	(0-81)	140	0 (0-0)	(0-46)	135	1 (1-3)	(0-72)
6th year	98	2 (1-5)	(0-103)	128	0 (0-1)	(0-95)	123	2 (1-3)	(0-54)
8th year	107	2 (1-4)	(0-54)	113	0 (0-0)	(0-20)	109	1 (1-3)	(0-52)
10th year	86	2 (1-4)	(0-45)	90	0 (0-0)	(0-22)	88	1 (1-2)	(0-41)
12th year	69	2 (1-4)	(0-31)	72	0 (0-0)	(0-14)	71	1 (0-3)	(0-21)
14th year	49	2 (1-5)	(0-20)	51	0 (0-1)	(0-14)	51	2 (1-4)	(0-19)
16th year	30	3 (1-6)	(0-24)	32	0 (0-1)	(0-4)	32	2 (1-4)	(0-19)
18th year	10	1 (0-2)	(0-16)	11	0 (0-0)	(0-0)	10	1 (0-2)	(0-16)

Patients had to be alive and have all data coverage data coverage for at least part of the year reported (row in table). Inpatient, outpatient and A&E data are available from 2000, 2003 and 2007 onwards till March 2018, respectively. Outpatient and A&E data were available for England patients only.
